# Supplementary material for: Cis-regulatory evolution integrated the Bric-à-brac transcription factors into a novel fruit fly gene regulatory network
Source: eLife. 2018 Jan 3;7:e32273. doi: 10.7554/eLife.32273 (PMC5752203; doi:10.7554/eLife.32273)
Supplement: Figure 2—source data 1. — The amino acid sequences for Drosophila (D.) melanogaster Bab1 and Bab2, D. ananassae Bab1 and Bab2, D. willistoni Bab1, D. mojavensis Bab2, Glossina morsitans Bab1 and Bab2, and Anopheles gambiae Bab were aligned using the Clusta Omega multiple sequence alignment program. The BTB and Conserved Domains for D. melanogaster Bab1 and Bab2 are respectively indicated by the maroon and blue background colors. Within the Conserved Domain the psq domain is indicated by the amino acids with yellow font color and the AT-hooks by amino acids with red font color. [file elife-32273-fig2-data1.docx]

**Figure 2-source data 1. Amino acid alignment for Bab homologs.** The amino acid sequences for *Drosophila* (*D.*) *melanogaster* Bab1 and Bab2, *D. ananassae* Bab1 and Bab2, *D. willistoni* Bab1, *D. mojavensis* Bab2, *Glossina morsitans* Bab1 and Bab2, and *Anopheles gambiae* Bab were aligned using the Clusta Omega multiple sequence alignment program. The BTB and Conserved Domains for *D. melanogaster* Bab1 and Bab2 are respectively indicated by the maroon and blue background colors. Within the Conserved Domain the psq domain is indicated by the amino acids with yellow font color and the AT-hooks by amino acids with red font color.

Gmor\bab2\GMOY011080-RA MNLTKNIMDFTVRARGTDL--VSLEATDELCAANHDS-------TVAQNSEAHVEFVAPA 51

Dmoj\bab2\XM_002007001.1 MDMTKDIMDFERKSLD-SSCGEQFEPSDYTMVNAELAKQAAQTAQAVDQVE--------- 50

Dmel\bab2\AAF47442.2 MDMTKQIVDFEIKSEL-IGEIDQFEASDYTMAPPEEPKMVEESPQLGHLEDQNRKYSPER 59

Dana\bab2\XP_001956730.1 MDMTKQIVDFELKSETEMAGVEQYEPSDYTMPGLLEPKEVVEP-ILEEPEESDVKSPQES 59

Agam\bab\AGAP006018-RA ------------------------MPSDTPPPSATSVSHP--SPAS-------------S 21

Gmor\bab1\GMOY011079 ------------------------MSSNNPLQTLPETGKKGKSPSI------------SN 24

Dwil\bab1\GK16863-PB ------------------------------MATLESTSQRNDRSET------------ET 18

Dmel\bab1\gbAAF47439.2 ------------------------------MAS--------AQAET------------NV 10

Dana\bab1\GF10081-PB ------------------------------MAT--------AQSET------------EA 10

Gmor\bab2\GMOY011080-RA GVELSRKT----AY---WEREKLRNPSTPEL—LENMDKSITKATTNCDLTPPPRPLTSS 102

Dmoj\bab2\XM_002007001.1 -----LDLPLELAKK—EEPEAQPEPMQQLKEENRAVAAEKPAMLNEQALTPPPRPLTSS 103

Dmel\bab2\AAF47442.2 EVEPTLQDPSEVVDQMQKDTESVGEVKSPEKDVETELVKSKASPMNDQALTPPPRPLTSS 119

Dana\bab2\XP_001956730.1 GMEASLEEPKENASHTDK---EMPEKTAAETDSDSVVAKAKASLLNDQALTPPPRPLTSS 116

Agam\bab\AGAP006018-RA HHDPN-------------DPNAP--PRD-------PVDRSG-------TGTPGPSDHPTG 52

Gmor\bab1\GMOY011079 LMDT------------------T---TD-------NKQPSR-------P-S-----S--- 40

Dwil\bab1\GK16863-PB GNETN-------------VEQAQ---SA-------QRQRSG-------GGG-----SNGG 43

Dmel\bab1\gbAAF47439.2 GLAS--------------EQG-P---VA-------QRQ-RK-------GTG-----SGAD 32

Dana\bab1\GF10081-PB GMPA--------------EQQAH---VT-------QRQRKG-------GTG-----SGAG 34

Gmor\bab2\GMOY011080-RA EVVGFTSTFEDPDIN-----VEATLALKKSRSLPASP---------------NHLANVRL 142

Dmoj\bab2\XM_002007001.1 EVVGHSEP---SDPE-----LQIQLTAKKSRSLPVSP---------------QPLVAHNL 140

Dmel\bab2\AAF47442.2 EVVGLRDP---EHTE-----LRMCLEAKKSRSLPVSP---------------QPQPNLKL 156

Dana\bab2\XP_001956730.1 EVVGLRDP---DDPE-----LRLRLEAKKSRSLPVSP---------------QPQQNLKI 153

Agam\bab\AGAP006018-RA GHLGHHQPPSSSSSSSSSSSSSSTSSSLSSLSLKRSLEEPLTTAKPSPPCSPLTMDHHHQ 112

Gmor\bab1\GMOY011079 VIPGILASLDSNSTSP----PITHVTKLESNTLRSRNEHIVEVSSVS--ETPSHVPTPHS 94

Dwil\bab1\GK16863-PB GGGGGITPTKSQPDSP----SNKTEDQK---SE-STPEQR------------RSPGGVRG 83

Dmel\bab1\gbAAF47439.2 -------SPKSNRSSP----TQQEEKRIKSEDR-TSPTG-----GAK--DEDKESQGHAV 73

Dana\bab1\GF10081-PB -------SPKSNRSSP----SQE-EKQAKSEDR-NSPAGPGSGGGVK--DEDKDNP--GA 77

.

Gmor\bab2\GMOY011080-RA TPLFAFTSRKAFEPPKIAETYAIN-KEPRTI-----KQHFETDKRNNQQFCLRWNNYQNN 196

Dmoj\bab2\XM_002007001.1 AAIGLFEFGKTVETPELKPKMNHKLLPPVNVGVAPRKVAPSAGGGDNQQFCLRWNNYQSN 200

Dmel\bab2\AAF47442.2 AGSALFEFGQRSSPVETKIKTNPETKPPR-----RKIVPPSGE---GQQFCLRWNNYQSN 208

Dana\bab2\XP_001956730.1 AASALFEFGRGSAPVESKIKSNPDVKPPR-----RRVAPPSGGGGDNQQFCLRWNNYQSN 208

Agam\bab\AGAP006018-RA HKA-ARQ-SRAASPAGRST---QQQASPSAP---GTGGSSSGGGGGGQQFCLRWNNYQTN 164

Gmor\bab1\GMOY011079 SSP-VCE-VPSSSPHSEQ------------------SSSGSASASAPQQFCLRWNNYQSN 134

Dwil\bab1\GK16863-PB ADG-AGS-SPVASPPARS------------------SSAASPNSNSAQQFCLRWNNYQTN 123

Dmel\bab1\gbAAF47439.2 AGG-GGS-SPVSSPQGRS------------------SSVASPSS-SSQQFCLRWNNYQTN 112

Dana\bab1\GF10081-PB SGV-GGS-SPVSSPQGRS------------------SSVASPSS-TSQQFCLRWNNYQTN 116

**BTB Domain Start**

Gmor\bab2\GMOY011080-RA LTNVFDELLQNESFVDVTLACEGQSIKAHKVVLSACSPYFQRLFYDNPCQHPIVIMRDVR 256

Dmoj\bab2\XM_002007001.1 LTNVFDELLQNESFVDVTLACDGQSIKAHKMVLSACSPYFQALFYDNPCQHPIIIMRDVN 260

Dmel\bab2\AAF47442.2 LTNVFDELLQSESFVDVTLSCEGHSIKAHKMVLSACSPYFQALFYDNPCQHPIIIMRDVS 268

Dana\bab2\XP_001956730.1 LTNVFDELLQSESFVDVTLACEGHSIKAHKMVLSACSPYFQALFYDNPCQHPIIIMRDVN 268

Agam\bab\AGAP006018-RA LTSVFDQLLQSESFVDVTLACDGQSMKAHKMVLSACSPYFQTLFFDNPCQHPIVIMRDVS 224

Gmor\bab1\GMOY011079 LTSVFDQLLQTESFVDVTLACDGHSIKAHKMVLSACSPYFQNLFFDTPCQHPIVIMRDVG 194

Dwil\bab1\GK16863-PB LTTIFDQLLQNECFVDVTLACDGRSLKAHKMVLSACSPYFQTLLAETPCQHPIVIMRDVN 183

Dmel\bab1\gbAAF47439.2 LTTIFDQLLQNECFVDVTLACDGRSMKAHKMVLSACSPYFQTLLAETPCQHPIVIMRDVN 172

Dana\bab1\GF10081-PB LTTIFDQLLQNECFVDVTLACDGRSMKAHKMVLSACSPYFQTLLAETPCQHPIVIMRDVN 176

**BTB Domain**

Gmor\bab2\GMOY011080-RA WQELKALMEFMYKGEINVSQDQINPLLKVAEMLKIRGLAEVNSTGT---AAAHPMVLEQ- 312

Dmoj\bab2\XM_002007001.1 WCDLKALVEFMYKGEINVCQDQINPLLKVAETLKIRGLAEVGASSTAAGLGAASMLPEQ- 319

Dmel\bab2\AAF47442.2 WSDLKALVEFMYKGEINVCQDQINPLLKVAETLKIRGLAEVSAGRGEGGAS------AL- 321

Dana\bab2\XP_001956730.1 WSDLKALVEFMYKGEINVCQDQINPLLKVAETLKIRGLAEVSAGRGDGGAS------AH- 321

Agam\bab\AGAP006018-RA WAELKAIVEFMYKGEINVSQDQIGPLLKVAEMLKIRGLADVSGDAGEPTGSRAEREAA-- 282

Gmor\bab1\GMOY011079 WCELKAIVDFMYKGEINVSQEQIGPLLRIAEMLKVRGLADVGNIESSTSDARPNELLEKH 254

Dwil\bab1\GK16863-PB WCDLKAIVEFMYRGEINVSQDQIGPLLRIAEMLKVRGLADVTHMEAAATAAAAAAAQQ-- 241

Dmel\bab1\gbAAF47439.2 WSDLKAIVEFMYRGEINVSQDQIGPLLRIAEMLKVRGLADVTHMEAATAAAAAASSER-- 230

Dana\bab1\GF10081-PB WSDLKAIVEFMYRGEINVSQDQIGPLLRIAEMLKVRGLADVTNMEAATAAAAAASESR-- 234

**BTB Domain End**

Gmor\bab2\GMOY011080-RA ------RM----------------AVYES--DEEKPVEND-EPY----------ADGEDI 337

Dmoj\bab2\XM_002007001.1 ------RM----------------SVYDD--EEDEDELAAAAALL-------NDE---DE 345

Dmel\bab2\AAF47442.2 ------PM----------------SAFDD--EDEEEELASATAIL-------QQDGDADP 350

Dana\bab2\XP_001956730.1 ------PM----------------SVYDD--EDDEEELAAAAAIL-------RQHDDADP 350

Agam\bab\AGAP006018-RA -------------------------GSRGPEELDREEHGKLLNPLAIVGSSLLANGAASA 317

Gmor\bab1\GMOY011079 DPSPQNLVSPKIPQHLSPITSREQSLYQSPDSGRAEQVQSFLSFT-EPNKKLR-LEKSAW 312

Dwil\bab1\GK16863-PB -----QVSSPK-----------ETQR----EAAEREAAEELLAFM-QPEKKLR-LGATDW 279

Dmel\bab1\gbAAF47439.2 -----MPSSPK-----------ESTSTSRTEHDREREAEELLAFM-QPEKKLR-T--SDW 270

Dana\bab1\GF10081-PB -----IPS-PK-----------EGTSASRT--ETEREAEELLAFM-QPEKKLR----TDW 270

Gmor\bab2\GMOY011080-RA T----------------NR-QK-----AKRPRINDTKK---------------------- 35

Dmoj\bab2\XM_002007001.1 ----------------EL-LK-----PKRARLLAKLRA---------------------- 362

Dmel\bab2\AAF47442.2 D----------------EE-MK-----AKRPRLL-------------------------- 362

Dana\bab2\XP_001956730.1 E----------------DE-LK-----AKRPRLL-------------------------- 362

Agam\bab\AGAP006018-RA AMAGGNGSNSTATSGSAAVQAAAAAAAAKKQRAGRDRD-----TT--------------- 357

Gmor\bab1\GMOY011079 DINSSS-SGNHPNSSSIELRLSPLPHGSLVTRNVRKRRWPSADAL---LNP--PSSPLSG 366

Dwil\bab1\GK16863-PB GDYG----------GGGELRLSPLERP-QVARNVRKRRWPSADTPMPIFNPP-SSSPLSS 327

Dmel\bab1\gbAAF47439.2 D--------------PAELRLSPLERQ--QGRNVRKRRWPSADTI---FNPPAPPSPLSS 311

Dana\bab1\GF10081-PB D--------------PAELRLSPLERQ--QGRNVRKRRWPSADTI---FNPPAPPSPLSS 311

Gmor\bab2\GMOY011080-RA --IRSKLDINFAPNRTRKRSRDGLLMDTDRFFSSSSHNDTYDYSKSSTGNFVEKNVPSSV 411

Dmoj\bab2\XM_002007001.1 --AETALDL---NQRQRKRSRDGSYATPSPLRSES--------------------PSSQL 397

Dmel\bab2\AAF47442.2 --PEGVLDL---NQRQRKRSRDGSYATPSPSLQGGESE------ISERGSSGTPGQSQSQ 411

Dana\bab2\XP_001956730.1 --ADGALDL---NQRQRKRSRDGSYATPSPSLQGGESE------TSERATPGN--QNQTQ 409

Agam\bab\AGAP006018-RA --KEHRMD---A--RLSEFARDLSRADPHIS--SRDISS-------VAAAAAAAAA-AAA 400

Gmor\bab1\GMOY011079 LIAAERAE---QEQ-ERERQREHILITPPIAITSSTTNSATHLTSSSLASNVQIEI-PSS 421

Dwil\bab1\GK16863-PB LIAAERLE---QEQKERERQRDCSLMTPPPKPSGA--------------TTPRRLT-EIH 369

Dmel\bab1\gbAAF47439.2 LIAAERME---LEQKERERQRDCSLMTPPPKPPMSSGS--------TVGATRRLET-AIH 359

Dana\bab1\GF10081-PB LIAAERLE---LEQKERERQRDCSLMTPPPKPPMSGAAVGVG----AAGAARRLET-AIH 363

Gmor\bab2\GMOY011080-RA PTAMTTSTIVRNPFASPNQTNQASDVDAKKAFLTSSLASSHSAASSDASSSSVTLPFRSM 471

Dmoj\bab2\XM_002007001.1 PLAMTTSTIVRNPFASPNPQTLPASSGSSSNSNSNNSSCN---NSSSNSSSTATAAAQPT 454

Dmel\bab2\AAF47442.2 PLAMTTSTIVRNPFASPNPQTLEGRNSAMNAV---------------------------- 443

Dana\bab2\XP_001956730.1 PLAMTTSTIVRNPFASPNPQSLQGSGASANER---------------------------- 441

Agam\bab\AGAP006018-RA GLA------V---GEWPLGA--AGLEAA-------------------------------- 417

Gmor\bab1\GMOY011079 SLT------LLPPARTPSGL--L----------T---PSPHLQISQHQSQ--LQQH---- 454

Dwil\bab1\GK16863-PB GLD------MPSPATTPAPA--IGLGRSARTL-A---PSPQQQHQQQRHS--SPAS---- 411

Dmel\bab1\gbAAF47439.2 ALD------MPSPAATPGPL--S---RSSRPH-S---QSPQQQQAQQQGQ--LPLP---- 398

Dana\bab1\GF10081-PB GLE------MPSPSATPGPL--S---RSSRTH-S---QSPQQQPGA--SQ--HPLP---- 400

Gmor\bab2\GMOY011080-RA TRSCSPSLA-----------------------AATHT-SREILALVSPTESQTSAHSRS- 506

Dmoj\bab2\XM_002007001.1 ATNCSSSSSAGVPSNGSSSAAYRSPPPPPPPPSSAHSNGSSAAGLSSPTGNKSSAAAAAA 514

Dmel\bab2\AAF47442.2 -------------------A--NQRKS-PAPTATGHSNGNSGAAMHSPPGG------VAV 475

Dana\bab2\XP_001956730.1 -------------------G--ASRGSPPPPSAHSNGSGNSGAALHSPPGS------SAA 474

Agam\bab\AGAP006018-RA ---------------------------AAAAV-------------------QASTPKSAR 431

Gmor\bab1\GMOY011079 -------HSSGQHS--------Q-RASPASSVASTQPSSVLSGPLTPSPANITSSNSGES 498

Dwil\bab1\GK16863-PB -------------S--------QSGASAAV--------HQA--------ASSAASSPAGG 434

Dmel\bab1\gbAAF47439.2 ---------LPLHP--------HHHASPAP-----HPSQTA--------GSAHHPASPAG 428

Dana\bab1\GF10081-PB ---------LPLHP--------HHHASPAP-----HPAQAA--------AAAHHPPSPAA 430

Gmor\bab2\GMOY011080-RA --------AGSVG------NA-------LADPNHPHHQAVAAAQHLAAQHQFHTAAQSHA 545

Dmoj\bab2\XM_002007001.1 QSQLPPHMAAAVAAAAHHASANVPPPPPGAAASMHHHAAAAAAQQLAAQHQLA---HSHA 571

Dmel\bab2\AAF47442.2 QSALPPHMAAI-----------VPPPP----SAMHHHA-----QQLAAQHQLA---HSHA 512

Dana\bab2\XP_001956730.1 QTALPPHMAAAVAAAAHHAAVVPPPPP----ASMHHHAAAAAAQQLAAQHQLA---HSHA 527

Agam\bab\AGAP006018-RA KRRWPSGERSSIGSPADSTPDQL---E--------------------VPSPIPPT----- 463

Gmor\bab1\GMOY011079 HHRFSMGSVQAAAMAAAAA-------A--------------------AHIDLTPA----- 526

Dwil\bab1\GK16863-PB DGRFPLGPAAAMAAAAMELSAL--GPP--------------------TEPRLPPP----- 467

Dmel\bab1\gbAAF47439.2 DSRFPLGPAAAMAA-ARELSGLGPGPS--------------------AEPRLPPP----- 462

Dana\bab1\GF10081-PB DSRFPLGPAAAMAA-AMELSGLGPGPP--------------------AEPRLPPP----- 464

Gmor\bab2\GMOY011080-RA VMASALGVSLAAVA-AGASS-----------------A---------SGITNNTGKITTG 578

Dmoj\bab2\XM_002007001.1 AMASVLGASLAAAA-A--G--------GAA-----APG-----------SAAGAGNAPSS 604

Dmel\bab2\AAF47442.2 MASALAAAAAGAGA-AGAGGAGSGSGSGAS-----APT---------GGTGVAGSGAGAA 557

Dana\bab2\XP_001956730.1 MASALAAAAAGSGA------AAAGSGAGAA-----APS---------SGAGAGGSASASS 567

Agam\bab\AGAP006018-RA --PSSLAQSSGGGG-GGGGGGGGGTGSGGGGGGSSNPLASFPLPPALDTAAMAMSSLSSS 520

Gmor\bab1\GMOY011079 --A---A--MGI---GGLPSATMPIGSGPTHHP--------------------------- 549

Dwil\bab1\GK16863-PB --P---PHHQGGST-----------------AS--------------------------- 478

Dmel\bab1\gbAAF47439.2 --P---PHHHGGGGVGGGGVGGGGAGGVGSGGG--------------------------- 490

Dana\bab1\GF10081-PB --P---SHHHGGGGGG-SGVGG---GGSGAGGG--------------------------- 488

.

Gmor\bab2\GMOY011080-RA PPSHHDDMEIKPEIAEMIREEERA------------------------------------ 602

Dmoj\bab2\XM_002007001.1 VGGHHDDMEIKPEIAEMIREEERA------------------------------------ 628

Dmel\bab2\AAF47442.2 VGSHHDDMEIKPEIAEMIREEERA------------------------------------ 581

Dana\bab2\XP_001956730.1 VGSHHDDMEIKPEIAEMIREEERA------------------------------------ 591

Agam\bab\AGAP006018-RA IANHPDDMEIKPGIAEMIREEERS------------------------------------ 544

Gmor\bab1\GMOY011079 -SSIADDLEIKPGIAEMIREEERENMAKTILMVCSGNGLCSSIAEAVMAEINEEAGVSHY 608

Dwil\bab1\GK16863-PB -SSLADDMEIKPGIAEMIREEERA------------------------------------ 501

Dmel\bab1\gbAAF47439.2 -SSLADDLEIKPGIAEMIREEERA------------------------------------ 513

Dana\bab1\GF10081-PB -SSLADDLEIKPGIAEMIREEERA------------------------------------ 511

**Bab Conserved Domain Start**

Gmor\bab2\GMOY011080-RA ------------------------------------------------------------ 602

Dmoj\bab2\XM_002007001.1 ------------------------------------------------------------ 628

Dmel\bab2\AAF47442.2 ------------------------------------------------------------ 581

Dana\bab2\XP_001956730.1 ------------------------------------------------------------ 591

Agam\bab\AGAP006018-RA ------------------------------------------------------------ 544

Gmor\bab1\GMOY011079 WQVDSNAVGGWNSDYSSDEGPISLCTDNETSPQDTLRRPELYRRLPALTFDDNNINGRAE 668

Dwil\bab1\GK16863-PB ------------------------------------------------------------ 501

Dmel\bab1\gbAAF47439.2 ------------------------------------------------------------ 513

Dana\bab1\GF10081-PB ------------------------------------------------------------ 511

Gmor\bab2\GMOY011080-RA -----KMIESGH--PWTAGT-----SSTSVTDSYQYQLQSMWQKCWNTNQQNLVQQLRFR 650

Dmoj\bab2\XM_002007001.1 -----KMIETSGH-AWMGAPAT---GASVAADSYQYQLQSMWQKCWNTNQQNLVQQLRFR 679

Dmel\bab2\AAF47442.2 -----KMIESGGHGGWMGAAAAATGAASVAADSYQYQLQSMWQKCWNTNQQNLVQQLRFR 636

Dana\bab2\XP_001956730.1 -----KMIESGGHGGWMGAAAAATGAASVAADSYQYQLQSMWQKCWNTNQQNLVQQLRFR 646

Agam\bab\AGAP006018-RA ----------------------------------------MWQKCWNS-Q-NLIHHLRFR 562

Gmor\bab1\GMOY011079 PNRAAKMLESSH--AWMSS------GASIAADSYQYQLQSMWQKCWNTNQ-SLMHHLRFR 719

Dwil\bab1\GK16863-PB -----KMMENSH--AWMGAT-----GSTL-ADSYQYQLQSMWQKCWNTNQ-NLMHHMRFR 547

Dmel\bab1\gbAAF47439.2 -----KMMENSH--AWMGAT-----GSTLAADSYQYQLQSMWQKCWNTNQ-NLMHHMRFR 560

Dana\bab1\GF10081-PB -----KMMENSH--AWMGAT-----GSTL-ADSYQYQLQSMWQKCWNTNQ-NLMHHMRFR 557

**Bab Conserved Domain**

Gmor\bab2\GMOY011080-RA ERGPLKSWRPEAMAEAIFSVLKEGLSLSQAARKYDIPYPTFVLYANRVHNMLGPSLDGGS 710

Dmoj\bab2\XM_002007001.1 ERGPLKSWRPEAMAEAIFSVLKEGLSLSQAARKYDIPYPTFVLYANRVHNMLGPSLDGGS 739

Dmel\bab2\AAF47442.2 ERGPLKSWRPEAMAEAIFSVLKEGLSLSQAARKFDIPYPTFVLYANRVHNMLGPSLDGGA 696

Dana\bab2\XP_001956730.1 ERGPLKSWRPEAMAEAIFSVLKEGLSLSQAARKYDIPYPTFVLYANRVHNMLGPSLDGGA 706

Agam\bab\AGAP006018-RA ERGPLKSWRPETMAEAIFSVLKEGLSLSQAARKYDIPYPTFVLYANRVHNMLGPSIDGGT 622

Gmor\bab1\GMOY011079 ERGPLKSWRPETMAEAIFSVLKEGLSLSQAARKYDIPYPTFVLYANRVHNMLGPSIDGGP 779

Dwil\bab1\GK16863-PB ERGPLKSWRPETMAEAIFSVLKEGLSLSQAARKYDIPYPTFVLYANRVHNMLGPSIDGGP 607

Dmel\bab1\gbAAF47439.2 ERGPLKSWRPETMAEAIFSVLKEGLSLSQAARKYDIPYPTFVLYANRVHNMLGPSIDGGP 620

Dana\bab1\GF10081-PB ERGPLKSWRPETMAEAIFSVLKEGLSLSQAARKYDIPYPTFVLYANRVHNMLGPSIDGGP 617

**psq Domain**

Gmor\bab2\GMOY011080-RA DPRPKARGRPQRILLGMWPDDLIRSVIKAVVFRDYREIKDELGG-----LSYVNGQPN-- 763

Dmoj\bab2\XM_002007001.1 DPRPKARGRPQRILLGMWPDELIRSVIKAVVFRDYREIKEDINA-----HPYANGQPH-- 792

Dmel\bab2\AAF47442.2 DPRPKARGRPQRILLGMWPEELIRSVIKAVVFRDYREIKEDMSA-----HQYANGQGH-- 749

Dana\bab2\XP_001956730.1 DPRPKARGRPQRILLGMWPEELIRSVIKAVVFRDYREIKEDMGA-----HQYANGQAH-- 759

Agam\bab\AGAP006018-RA DLRPKGRGRPQRILLGIWPDDHIKGVIKSVVFRDAKDMKEE---------PMMYGRHSP- 672

Gmor\bab1\GMOY011079 DLRPKGRGRPQRILLGIWPDEHIKGVIKTVVFRDAKDL-KEETF-----AHLSYGRHSPV 833

Dwil\bab1\GK16863-PB DLRPKGRGRPQRILLGIWPDEHIKGVIKTVVFRDATKELKDDSALGLGGHMPPYGRHS-- 665

Dmel\bab1\gbAAF47439.2 DLRPKGRGRPQRILLGIWPDEHIKGVIKTVVFRDTKD-IKD---ESLAAHMPPYGRHSPA 676

Dana\bab1\GF10081-PB DLRPKGRGRPQRILLGIWPDEHIKGVIKTVVFRDAKD-LKD---DSIGSHLPPYGRHS-- 671

**AT Hook** **Bab Conserved Domain End**

Gmor\bab2\GMOY011080-RA -----VPPHFSNPNT---------IITNGMHNAA---KLAVQN---TILASQES------ 797

Dmoj\bab2\XM_002007001.1 -----GA-HYGSNSA---------AAANGYHSAT------------VKMAPPDA------ 819

Dmel\bab2\AAF47442.2 -----GT-YIGGGT-----------TTNGYHSAAAA-KLAAQN--A-ALAPPDA------ 782

Dana\bab2\XP_001956730.1 -----GS-HFGPGS-----------ASNGYHSAAAA-KMAAQN--AAALAPPDS------ 793

Agam\bab\AGAP006018-RA FPFQDNPLSYGPTAPN------------GQLPS-------------VATGT-NVPDGMSQ 706

Gmor\bab1\GMOY011079 FSFQESTLNYGGPSS---------QCANGM--------------------PAPASDQMSQ 864

Dwil\bab1\GK16863-PB ----DMSLSYPGAASAA-----ALACSNGMGGGGAGVGVGVGGGPSVVGGPGPDQGQMSQ 716

Dmel\bab1\gbAAF47439.2 FPLQDLPLSYPGASGALAGAPSSMACPNGSGP---QTGVGVAG-----------EQHMSQ 722

Dana\bab1\GF10081-PB ----DLPLSYPGASGALAGPPSSLACPNGSGP---QGGV--GG-----------EPHMSQ 711

Gmor\bab2\GMOY011080-RA ----SSPLNSMTENFRRHIISQQQQ--------------------HSPVSQNMNLYKSPA 833

Dmoj\bab2\XM_002007001.1 ----SNPLSTMTETLRRQILSQQQQQQQQQQQQQ-----HQQHQQQSPHMQSMNMYKSPA 870

Dmel\bab2\AAF47442.2 ----GSPLSSMTETLRRQILSQQQQHQQHHQQQAHHQQQPSHHQQQSPHAQSMNMYKSPA 838

Dana\bab2\XP_001956730.1 ----GSPLSSMTETLRRQILSQQQQQQHHQQQQSHHQQQPSHHQQQSPHGQSMNMYKSPA 849

Agam\bab\AGAP006018-RA DALTAATVA----AVRQQMCNMVAAAQH-----------HPDAAN----LVAAAGFNLPS 747

Gmor\bab1\GMOY011079 EAT-AAAVAAVAHNFRQQM-QMAAAAQH-----------QQHSENIG----AASLFNLPP 907

Dwil\bab1\GK16863-PB -ET-AAAVAAVAHNIRQQM-QMAAAVQQ-----------QHQHGEAGP---PPGLFNLPP 759

Dmel\bab1\gbAAF47439.2 -ET-AAAVAAVAHNIRQQM-QMAA--------------------------VPPGLFNLPP 753

Dana\bab1\GF10081-PB -ET-AAAVAAVAHNIRQQM-QMAAAVQH-------------QHGEAGPPPVPPGLFNLPP 755

Gmor\bab2\GMOY011080-RA YLQRSEMSEQSPDL------------LSKQ--------MSERRSAENLADLS------KL 867

Dmoj\bab2\XM_002007001.1 YLQRSEIEDQVSAAAAVAAA--K-----HQ--------QNERRGSENLPDLS------AL 909

Dmel\bab2\AAF47442.2 YLQRSEIEDQVSAAAAVAAA--AAKHQ-QQ--------QGERRGSENLPDLS------AL 881

Dana\bab2\XP_001956730.1 YLQRSEIEDQVSAAAAVAAA--AAKHQQQQ--------QGDRRGSENLPDLS------AL 893

Agam\bab\AGAP006018-RA HCGTPPNLSMHPAAAAAAAAAAAASNASAAGGPSGGGGGGGSSGAIPLPKMG-SPAVPST 806

Gmor\bab1\GMOY011079 HLVSAAG--------------------PAGAGPIVGAPGP---GSIVLPKPSISPALSTT 944

Dwil\bab1\GK16863-PB HLAGSG--------------------------PVL---GR---GS------SISPALSS- 780

Dmel\bab1\gbAAF47439.2 HPGVGG-----------------------GVGNVPGAAGG---RA------SISPALSS- 780

Dana\bab1\GF10081-PB HPGVGS-------------------------VPVPGSGGG---RA------SISPALSS- 780

Gmor\bab2\GMOY011080-RA GLM--------NLSGLNALPPSGPCPN---------------------------QSIHHN 892

Dmoj\bab2\XM_002007001.1 GLI--------GLPGLNVMPTQQQPGG--------------HQRGGPGSGGAAAGGLHPN 947

Dmel\bab2\AAF47442.2 GLM--------GLPGLNVMPSRGSGGG-------------------------SGGAAPNS 908

Dana\bab2\XP_001956730.1 GLM--------GLPGLNVMPSRGSAGG-------------------------AGGAAPNS 920

Agam\bab\AGAP006018-RA GHGNNNGGSGAGIQMPRLG---SPAGSSGLAKEHELQHHGGGGGGGGGLGGGSGGGMSRA 863

Gmor\bab1\GMOY011079 ---SNSGG-GSAVVGPRHAP--SPCGPTL-------------------------PGMH-Q 972

Dwil\bab1\GK16863-PB ------------GSGPRHAPPSSPCGPAG--------------------------LMP-N 801

Dmel\bab1\gbAAF47439.2 ------------GSGPRHAP--SPCGPAG--------------------------LLP-N 799

Dana\bab1\GF10081-PB ------------GSGPRHAP--SPCGPAG--------------------------LLP-N 799

.

Gmor\bab2\GMOY011080-RA ANTYT-----------------------------IEREMECSRE-----KERDHKLKEA- 917

Dmoj\bab2\XM_002007001.1 AASYARE-----------------------LSRERERERERERE-----MSRDRELKEA- 978

Dmel\bab2\AAF47442.2 AASYARE-----------------------LSRERERDRE--RE-----RER-------- 930

Dana\bab2\XP_001956730.1 AASYARE-----------------------LSRERERDRERERE-----RER-------- 944

Agam\bab\AGAP006018-RA TPPGARDRAMTARSNLAAGETGRSSSSAGSIHRSSP-SS--SAGSSLN-HQHPAHLSHPH 919

Gmor\bab1\GMOY011079 LPPGMA----------------------VALHMVGG-TGRCETATMLNQQQHQHQLQQLH 1009

Dwil\bab1\GK16863-PB LPPSMA----------------------VALHRGDP-AA---AQALIKQQQF-------- 827

Dmel\bab1\gbAAF47439.2 LPPSMA----------------------VALHHQQ------------QQQAAHHHMQQLH 825

Dana\bab1\GF10081-PB LPPSMA----------------------VALHHQQQ-QA---AHHQQQQQAAHHHLQQLH 833

Gmor\bab2\GMOY011080-RA -----------------------IQARQFGNCSRGSATGASDGQ--------KQAPPSC- 945

Dmoj\bab2\XM_002007001.1 -----------------------MHARQYGNQSRGSNSSAGSKS----------AA--S 1002

Dmel\bab2\AAF47442.2 -----------------------ELSRQYGSQSRGSSSGSGSAK--------SLTA--S- 956

Dana\bab2\XP_001956730.1 -----------------------ELSRQYGSQSRGSSSGSGSAK--------SLSA--S 970

Agam\bab\AGAP006018-RA HQQQHHHQHHHQPHHGHAHHLPH-------HNPLSHLVGSGGASG--ALSITKLGSPGSA 970

Gmor\bab1\GMOY011079 MQQQHAI--HQQQQQQ----QKHHQQMIFGASSISHPSISTATTAAPASSQHSISTTISA 1063

Dwil\bab1\GK16863-PB ------------------------------------------------------------ 827

Dmel\bab1\gbAAF47439.2 LQQQQAHLHHHQQQQ--QQQQQQHHQGG--HQV-AH-----KS-GFGASSSSSAS---S- 870

Dana\bab1\GF10081-PB LQQQQQQAAHLHHQQQQQHQQHHHHQVP--HKS-VGGSGGGAG-GFGAS---------S- 879

Gmor\bab2\GMOY011080-RA ----FSTGPSSP---YP----------YFKNKDHAIQYAYNKKFLENLPPGIDFEAIANG 988

Dmoj\bab2\XM_002007001.1 ----SRPGAAASPYSAH----------YAKHAKEHPSYAYNKRFLESLPAGIDFEAIANG 1048

Dmel\bab2\AAF47442.2 ----QRPGAASPYSAAH----------YAK----HQASAYNKRFLESLPAGIDLEAFANG 998

Dana\bab2\XP_001956730.1 ----QRPGAASPYSA------------YAK----HQASAYNKRFLESLPAGIDFEAIANG 1010

Agam\bab\AGAP006018-RA HDLRISNSPDESPLASPIGLAMEPAVNLALGAGGTQPGPEDVRLHVPPPYGSKPPSRGGG 1030

Gmor\bab1\GMOY011079 PQTKFSSSSISPSLE-----------------RQRQSTPGNTALRSSL---------TDL 1097

Dwil\bab1\GK16863-PB ------HQQLDKPKT-----------------KGSPMRSETPRLHSPL---------TDL 855

Dmel\bab1\gbAAF47439.2 ----SSMGQHHAPKA-----------------KSSPLRSETPRLHSPL---------GDL 900

Dana\bab1\GF10081-PB ----SSQSSTHPSKA-----------------KGSPLRSETPRLHSPL---------GDL 909

Gmor\bab2\GMOY011080-RA LF---QKSAVKSPRFEDLFSGQ--DGNELLPTN--------------------------- 1016

Dmoj\bab2\XM_002007001.1 LL---QKSVNKSPRFEDFFPG--QDMSELFGSADASAGSGAGSAAA----------AAAA 1093

Dmel\bab2\AAF47442.2 LL---QKSVNKSPRFEDFFPGPGQDMSELFANPDASAAAAAAA-------------YAPP 1042

Dana\bab2\XP_001956730.1 LL---QKSVNKSPRFEDFFPGPGQDMSELFANPDAAAAAAAAA-------------AYAP 1054

Agam\bab\AGAP006018-RA APSTGYTSNSSPPRPEHLFQD--QDIAALVATTRA------------------------- 1063

Gmor\bab1\GMOY011079 ALDFGYSKSTQSFSPSRLFPD---DLADLVGTSPSSSTSKKPSTLADTSSTITVTKSNTP 1154

Dwil\bab1\GK16863-PB GLEMS--SYKRDYSPSRLFAD---DLAELVGAGGGGAASVSSSSSATTTAAAAAAAAAAA 910

Dmel\bab1\gbAAF47439.2 GLDMA--SYKREFSPSRLFAE---DLAELVGASVSSS------SSSAAAATAPPERSAGA 949

Dana\bab1\GF10081-PB GLDMA--GYKRDFSPSRLFAE---DLAELVGASVSSS------SSSQAAAAGAVAGADRS 958

Gmor\bab2\GMOY011080-RA ---------ETGVATAFPSQRDSNLMQIKLEQQQITEMQNEG 1049

Dmoj\bab2\XM_002007001.1 --------AAAAAAYAPPGMRESPLMKIKLEQQQAAELPHED 1127

Dmel\bab2\AAF47442.2 --------G---------AIRESPLMKIKLEQQHATELPHED 1067

Dana\bab2\XP_001956730.1 --------GAVGGAGGGPGIRESPLMKIKLEQQHATELPHED 1088

Agam\bab\AGAP006018-RA -------ACPPSRVPDYKDTAVRPTASIKVEPLTECRGD--- 1095

Gmor\bab1\GMOY011079 SEILTSVACTST--TTSTTSNKSSSNSVKLEPITTSSE---- 1190

Dwil\bab1\GK16863-PB AA--AAAAVAAA--TSGTSGSGGDASGIKVEPITTTSGE--- 945

Dmel\bab1\gbAAF47439.2 ------ASAATG--ADAP--SSSSSGGIKVEPITTTSE---- 977

Dana\bab1\GF10081-PB ------GAGSSG--VDAPSSSSAAPGAIKVEPITTTSE---- 988
