# Supplementary material for: Cis-regulatory evolution integrated the Bric-à-brac transcription factors into a novel fruit fly gene regulatory network
Source: eLife. 2018 Jan 3;7:e32273. doi: 10.7554/eLife.32273 (PMC5752203; doi:10.7554/eLife.32273)
Supplement: Figure 2—source data 2. — Purple background with white letters indicate the AscI (GGCGCGCC) and SbfI (CCTGCAGG) restriction enzymes sites that were appended to primers for cloning CRE versions into matching sites in the S3aG reporter transgene vector. Maroon background and white letters indicate sequences that comprise scanning mutations. The lower case nucleotide letters indicate the non-complementary transversions. The yellow background with black letters indicates the Abd-B sites identified in Jeong et al. (2006) which were not mutated in this study. The blue background with white letters indicate the Bab-bound sequences identified in this study. The gray background with bolded black letters indicate the regions to which the BE2.5 Fwd and BE3.5 Rvs primers (reverse complement of that highlighted) were designed to initially amplify the wild-type CRE sequence. [file elife-32273-fig2-data2.doc]

**Figure 2-source data 2. Sequence alignment of the yBE0.6 with scanning mutant versions.** Purple background with white letters indicate the AscI (GGCGCGCC) and SbfI (CCTGCAGG) restriction enzymes sites that were appended to primers for cloning CRE versions into matching sites in the S3aG reporter transgene vector. Maroon background and white letters indicate sequences that comprise scanning mutations. The lower case nucleotide letters indicate the non-complementary transversions. The yellow background with black letters indicates the Abd-B sites identified in Jeong et al. (2006) which were not mutated in this study. The blue background with white letters indicate the Bab-bound sequences identified in this study. The gray background with bolded black letters indicate the regions to which the BE2.5 Fwd and BE3.5 Rvs primers (reverse complement of that highlighted) were designed to initially amplify the wild type CRE sequence.

Alignment (DIALIGN format):

===========================

**BE2.5 Fwd**

yBE0.6 1 GGCGCGCC**CT GTGGGTGCAA TGATTTAGAA TG**CGGGCAAG GGATCAAGTT

SM1 1 GGCGCGCCaT tTtGtTtCcA gGcTgTcGcA gGaGtGaAcG tGcTaAcGgT

SM2 1 GGCGCGCCCT GTGGGTGCAA TGATTTAGAA TGCGGGCAAG GGATCAAGTT

SM3 1 GGCGCGCCCT GTGGGTGCAA TGATTTAGAA TGCGGGCAAG GGATCAAGTT

SM4 1 GGCGCGCCCT GTGGGTGCAA TGATTTAGAA TGCGGGCAAG GGATCAAGTT

SM5 1 GGCGCGCCCT GTGGGTGCAA TGATTTAGAA TGCGGGCAAG GGATCAAGTT

SM6 1 GGCGCGCCCT GTGGGTGCAA TGATTTAGAA TGCGGGCAAG GGATCAAGTT

SM7 1 GGCGCGCCCT GTGGGTGCAA TGATTTAGAA TGCGGGCAAG GGATCAAGTT

SM8 1 GGCGCGCCCT GTGGGTGCAA TGATTTAGAA TGCGGGCAAG GGATCAAGTT

SM9 1 GGCGCGCCCT GTGGGTGCAA TGATTTAGAA TGCGGGCAAG GGATCAAGTT

SM10 1 GGCGCGCCCT GTGGGTGCAA TGATTTAGAA TGCGGGCAAG GGATCAAGTT

yBE0.6 51 GAACCACTTC TAAGAAAAAA TAGCATTGCA TAAATGATAT AGAGTCCAAA

SM1 51 tAcCaAaTgC gAcGcAcAcA gAtCcTgGCA TAAATGATAT AGAGTCCAAA

SM2 51 GAACCACTTC TAAGAAAAcA gAtCcTgGaA gAcAgGcTcT cGcGgCaAcA

SM3 51 GAACCACTTC TAAGAAAAAA TAGCATTGCA TAAATGATAT AGAGTCCAAA

SM4 51 GAACCACTTC TAAGAAAAAA TAGCATTGCA TAAATGATAT AGAGTCCAAA

SM5 51 GAACCACTTC TAAGAAAAAA TAGCATTGCA TAAATGATAT AGAGTCCAAA

SM6 51 GAACCACTTC TAAGAAAAAA TAGCATTGCA TAAATGATAT AGAGTCCAAA

SM7 51 GAACCACTTC TAAGAAAAAA TAGCATTGCA TAAATGATAT AGAGTCCAAA

SM8 51 GAACCACTTC TAAGAAAAAA TAGCATTGCA TAAATGATAT AGAGTCCAAA

SM9 51 GAACCACTTC TAAGAAAAAA TAGCATTGCA TAAATGATAT AGAGTCCAAA

SM10 51 GAACCACTTC TAAGAAAAAA TAGCATTGCA TAAATGATAT AGAGTCCAAA

yBE0.6 101 AACTACACAA ATTCAATAGC AGTAATGGTT ACATTAGCTT TGAAATTGTT

SM1 101 AACTACACAA ATTCAATAGC AGTAATGGTT ACATTAGCTT TGAAATTGTT

SM2 101 cAaTcCcCcA cTgCcAgAtC cGgAcTtGgT cCcTgAtCTT TGAAATTGTT

SM3 101 AACTACACAA ATTCAATAGC AGTAATGGgT cCcTgAtCgT gGcAcTgGgT

SM4 101 AACTACACAA ATTCAATAGC AGTAATGGTT ACATTAGCTT TGAAATTGTT

SM5 101 AACTACACAA ATTCAATAGC AGTAATGGTT ACATTAGCTT TGAAATTGTT

SM6 101 AACTACACAA ATTCAATAGC AGTAATGGTT ACATTAGCTT TGAAATTGTT

SM7 101 AACTACACAA ATTCAATAGC AGTAATGGTT ACATTAGCTT TGAAATTGTT

SM8 101 AACTACACAA ATTCAATAGC AGTAATGGTT ACATTAGCTT TGAAATTGTT

SM9 101 AACTACACAA ATTCAATAGC AGTAATGGTT ACATTAGCTT TGAAATTGTT

SM10 101 AACTACACAA ATTCAATAGC AGTAATGGTT ACATTAGCTT TGAAATTGTT

yBE0.6 151 TTTAGACATC CGAAGAAATA AGATTAAATT TAAACGGCAT TCTTTAATTT

SM1 151 TTTAGACATC CGAAGAAATA AGATTAAATT TAAACGGCAT TCTTTAATTT

SM2 151 TTTAGACATC CGAAGAAATA AGATTAAATT TAAACGGCAT TCTTTAATTT

SM3 151 gTgAtAaAgC aGcAtAcAgA cGcTgAcAgT gAcAaGtCcT gCgTgAcTTT

SM4 151 TTTAGACATC CGAAGAAATA AGATTAAATT TAAACGGCcT gCgTgAcTgT

SM5 151 TTTAGACATC CGAAGAAATA AGATTAAATT TAAACGGCAT TCTTTAATTT

SM6 151 TTTAGACATC CGAAGAAATA AGATTAAATT TAAACGGCAT TCTTTAATTT

SM7 151 TTTAGACATC CGAAGAAATA AGATTAAATT TAAACGGCAT TCTTTAATTT

SM8 151 TTTAGACATC CGAAGAAATA AGATTAAATT TAAACGGCAT TCTTTAATTT

SM9 151 TTTAGACATC CGAAGAAATA AGATTAAATT TAAACGGCAT TCTTTAATTT

SM10 151 TTTAGACATC CGAAGAAATA AGATTAAATT TAAACGGCAT TCTTTAATTT

**Bab1 bound region**

yBE0.6 201 GTATTTTAAT ATTTTGAGAG GTTTTCCTTA TTTAAAGTGT AGATTATTGA

SM1 201 GTATTTTAAT ATTTTGAGAG GTTTTCCTTA TTTAAAGTGT AGATTATTGA

SM2 201 GTATTTTAAT ATTTTGAGAG GTTTTCCTTA TTTAAAGTGT AGATTATTGA

SM3 201 GTATTTTAAT ATTTTGAGAG GTTTTCCTTA TTTAAAGTGT AGATTATTGA

SM4 201 tTcTgTgAcT cTgTgGcGcG tTgTgCaTgA gTgAcAtTtT cGcTgAgTtA

SM5 201 GTATTTTAAT ATTTTGAGAG GTTTTCCTTA TTTAAAGTGT AGATTATTtA

SM6 201 GTATTTTAAT ATTTTGAGAG GTTTTCCTTA TTTAAAGTGT AGATTATTGA

SM7 201 GTATTTTAAT ATTTTGAGAG GTTTTCCTTA TTTAAAGTGT AGATTATTGA

SM8 201 GTATTTTAAT ATTTTGAGAG GTTTTCCTTA TTTAAAGTGT AGATTATTGA

SM9 201 GTATTTTAAT ATTTTGAGAG GTTTTCCTTA TTTAAAGTGT AGATTATTGA

SM10 201 GTATTTTAAT ATTTTGAGAG GTTTTCCTTA TTTAAAGTGT AGATTATTGA

yBE0.6 251 GGATTAATGC AAACCACTTT ATCTGCGGAG GTCGTAAAAC GTATTTTTAC

SM1 251 GGATTAATGC AAACCACTTT ATCTGCGGAG GTCGTAAAAC GTATTTTTAC

SM2 251 GGATTAATGC AAACCACTTT ATCTGCGGAG GTCGTAAAAC GTATTTTTAC

SM3 251 GGATTAATGC AAACCACTTT ATCTGCGGAG GTCGTAAAAC GTATTTTTAC

SM4 251 tGcTgAcTGC AAACCACTTT ATCTGCGGAG GTCGTAAAAC GTATTTTTAC

SM5 251 tGcTgAcTtC cAcCaAaTgT cTaTtCtGcG t**TCGTAAAA**a GgAgTgTgAa

SM6 251 GGATTAATGC AAACCACTTT ATCTGCGGAG GTCGTAAAAC GTATTTTTAC

SM7 251 GGATTAATGC AAACCACTTT ATCTGCGGAG GTCGTAAAAC GTATTTTTAC

SM8 251 GGATTAATGC AAACCACTTT ATCTGCGGAG GTCGTAAAAC GTATTTTTAC

SM9 251 GGATTAATGC AAACCACTTT ATCTGCGGAG GTCGTAAAAC GTATTTTTAC

SM10 251 GGATTAATGC AAACCACTTT ATCTGCGGAG GTCGTAAAAC GTATTTTTAC

yBE0.6 301 CCATTTGCAT GTTTATTATG CGTGTGGCTG GTTGTATTAC TTTACTTAAG

SM1 301 CCATTTGCAT GTTTATTATG CGTGTGGCTG GTTGTATTAC TTTACTTAAG

SM2 301 CCATTTGCAT GTTTATTATG CGTGTGGCTG GTTGTATTAC TTTACTTAAG

SM3 301 CCATTTGCAT GTTTATTATG CGTGTGGCTG GTTGTATTAC TTTACTTAAG

SM4 301 CCATTTGCAT GTTTATTATG CGTGTGGCTG GTTGTATTAC TTTACTTAAG

SM5 301 CaAgTgGaAg GgTgAgTcTG CGTGTGGCTG GTTGTATTAC TTTACTTAAG

SM6 301 CCATTTGCcT gT**TTATTATG** aGgGgGtCgG tTgGgAgTcC gTgAaTgAcG

SM7 301 CCATTTGCAT GTTTATTATG CGTGTGGCTG GTTGTATTAC TTTACTTAAG

SM8 301 CCATTTGCAT GTTTATTATG CGTGTGGCTG GTTGTATTAC TTTACTTAAG

SM9 301 CCATTTGCAT GTTTATTATG CGTGTGGCTG GTTGTATTAC TTTACTTAAG

SM10 301 CCATTTGCAT GTTTATTATG CGTGTGGCTG GTTGTATTAC TTTACTTAAG

yBE0.6 351 TTTTGCAATT TTTTCTTTAG CAAGCAGGTG CATTTGGGCC AAGAGATATA

SM1 351 TTTTGCAATT TTTTCTTTAG CAAGCAGGTG CATTTGGGCC AAGAGATATA

SM2 351 TTTTGCAATT TTTTCTTTAG CAAGCAGGTG CATTTGGGCC AAGAGATATA

SM3 351 TTTTGCAATT TTTTCTTTAG CAAGCAGGTG CATTTGGGCC AAGAGATATA

SM4 351 TTTTGCAATT TTTTCTTTAG CAAGCAGGTG CATTTGGGCC AAGAGATATA

SM5 351 TTTTGCAATT TTTTCTTTAG CAAGCAGGTG CATTTGGGCC AAGAGATATA

SM6 351 gTgTtCcAgT gTgTaTgTcG aAcGaAtGTG CATTTGGGCC AAGAGATATA

SM7 351 TTTTGCAATT TTTTCTTTaG aAcGaAtGgG aAgTgGtGaC cAtAtAgAgA

SM8 351 TTTTGCAATT TTTTCTTTAG CAAGCAGGTG CATTTGGGCC AAGAGATATA

SM9 351 TTTTGCAATT TTTTCTTTAG CAAGCAGGTG CATTTGGGCC AAGAGATATA

SM10 351 TTTTGCAATT TTTTCTTTAG CAAGCAGGTG CATTTGGGCC AAGAGATATA

yBE0.6 401 TGCGATCGCT TTCGGTTCGA ATTTTTAACA TTTACTTGCG GCGATGGTCA

SM1 401 TGCGATCGCT TTCGGTTCGA ATTTTTAACA TTTACTTGCG GCGATGGTCA

SM2 401 TGCGATCGCT TTCGGTTCGA ATTTTTAACA TTTACTTGCG GCGATGGTCA

SM3 401 TGCGATCGCT TTCGGTTCGA ATTTTTAACA TTTACTTGCG GCGATGGTCA

SM4 401 TGCGATCGCT TTCGGTTCGA ATTTTTAACA TTTACTTGCG GCGATGGTCA

SM5 401 TGCGATCGCT TTCGGTTCGA ATTTTTAACA TTTACTTGCG GCGATGGTCA

SM6 401 TGCGATCGCT TTCGGTTCGA ATTTTTAACA TTTACTTGCG GCGATGGTCA

SM7 401 gGaGcTaGaT gTaGtTgCtA cTgTgtcAaA gTgAaTgGCG GCGATGGTCA

SM8 401 TGCGATCGCT TTCGGTTCGA ATTTTTAAaA gTgAaTgGaG tCtAgGtTaA

SM9 401 TGCGATCGCT TTCGGTTCGA ATTTTTAACA TTTACTTGCG GCGATGGTCA

SM10 401 TGCGATCGCT TTCGGTTCGA ATTTTTAACA TTTACTTGCG GCGATGGTCA

yBE0.6 451 TTAGAGCATT ACCCACTTAG GGCACCCCCA ACATCCAGTT GATTTTCAGG

SM1 451 TTAGAGCATT ACCCACTTAG GGCACCCCCA ACATCCAGTT GATTTTCAGG

SM2 451 TTAGAGCATT ACCCACTTAG GGCACCCCCA ACATCCAGTT GATTTTCAGG

SM3 451 TTAGAGCATT ACCCACTTAG GGCACCCCCA ACATCCAGTT GATTTTCAGG

SM4 451 TTAGAGCATT ACCCACTTAG GGCACCCCCA ACATCCAGTT GATTTTCAGG

SM5 451 TTAGAGCATT ACCCACTTAG GGCACCCCCA ACATCCAGTT GATTTTCAGG

SM6 451 TTAGAGCATT ACCCACTTAG GGCACCCCCA ACATCCAGTT GATTTTCAGG

SM7 451 TTAGAGCATT ACCCACTTAG GGCACCCCCA ACATCCAGTT GATTTTCAGG

SM8 451 gTcGcGaAgT cCaCcCgTcG tGaAaCaCaA cCcTaCcGgT tAgTgTaAGG

SM9 451 TTAGAGCATT ACCCACTTAG GGCACCCCCA ACATCCAGgT tAgTgTaAtG

SM10 451 TTAGAGCATT ACCCACTTAG GGCACCCCCA ACATCCAGTT GATTTTCAGG

yBE0.6 501 GACCACAATA TTTTAAATAA CAGCTAGTGG AATTACCTAA AAGCGCTTTC

SM1 501 GACCACAATA TTTTAAATAA CAGCTAGTGG AATTACCTAA AAGCGCTTTC

SM2 501 GACCACAATA TTTTAAATAA CAGCTAGTGG AATTACCTAA AAGCGCTTTC

SM3 501 GACCACAATA TTTTAAATAA CAGCTAGTGG AATTACCTAA AAGCGCTTTC

SM4 501 GACCACAATA TTTTAAATAA CAGCTAGTGG AATTACCTAA AAGCGCTTTC

SM5 501 GACCACAATA TTTTAAATAA CAGCTAGTGG AATTACCTAA AAGCGCTTTC

SM6 501 GACCACAATA TTTTAAATAA CAGCTAGTGG AATTACCTAA AAGCGCTTTC

SM7 501 GACCACAATA TTTTAAATAA CAGCTAGTGG AATTACCTAA AAGCGCTTTC

SM8 501 GACCACAATA TTTTAAATAA CAGCTAGTGG AATTACCTAA AAGCGCTTTC

SM9 501 tAaCcCcAgA gTgTcAcTcA aAtCgAtTtG cAgTcCaTcA cAtCtCgTgC

SM10 501 GACCACAATA TTTTAAATAA CAGCTAGTGG AATTACCTAA AAGCGCTTgC

**Bab1 bound region**

yBE0.6 551 GTCCCTTTTG AAATTTTATG TAACACTCAA TTATATTTAT GTATATGTAT

SM1 551 GTCCCTTTTG AAATTTTATG TAACACTCAA TTATATTTAT GTATATGTAT

SM2 551 GTCCCTTTTG AAATTTTATG TAACACTCAA TTATATTTAT GTATATGTAT

SM3 551 GTCCCTTTTG AAATTTTATG TAACACTCAA TTATATTTAT GTATATGTAT

SM4 551 GTCCCTTTTG AAATTTTATG TAACACTCAA TTATATTTAT GTATATGTAT

SM5 551 GTCCCTTTTG AAATTTTATG TAACACTCAA TTATATTTAT GTATATGTAT

SM6 551 GTCCCTTTTG AAATTTTATG TAACACTCAA TTATATTTAT GTATATGTAT

SM7 551 GTCCCTTTTG AAATTTTATG TAACACTCAA TTATATTTAT GTATATGTAT

SM8 551 GTCCCTTTTG AAATTTTATG TAACACTCAA TTATATTTAT GTATATGTAT

SM9 551 tTaCaTgTTG AAATTTTATG TAACACTCAA TTATATTTAT GTATATGTAT

SM10 551 tTaCaTgTgG cAcTgTgAgG gAcCcCgCcA gTcTcTgTcT tTcTcTtTcT

yBE0.6 601 **GCTCAAAATC ACCTGCCAAT AAC**CCTGCAG G

SM1 601 GCTCAAAATC ACCTGCCAAT AACCCTGCAG G

SM2 601 GCTCAAAATC ACCTGCCAAT AACCCTGCAG G

SM3 601 GCTCAAAATC ACCTGCCAAT AACCCTGCAG G

SM4 601 GCTCAAAATC ACCTGCCAAT AACCCTGCAG G

SM5 601 GCTCAAAATC ACCTGCCAAT AACCCTGCAG G

SM6 601 GCTCAAAATC ACCTGCCAAT AACCCTGCAG G

SM7 601 GCTCAAAATC ACCTGCCAAT AACCCTGCAG G

SM8 601 GCTCAAAATC ACCTGCCAAT AACCCTGCAG G

SM9 601 GCTCAAAATC ACCTGCCAAT AACCCTGCAG G

SM10 601 tCgCcAcAgC cCaTtCaAcT cAaCCTGCAG G

**BE3.5 Rvs**
