# Supplementary material for: Cis-regulatory evolution integrated the Bric-à-brac transcription factors into a novel fruit fly gene regulatory network
Source: eLife. 2018 Jan 3;7:e32273. doi: 10.7554/eLife.32273 (PMC5752203; doi:10.7554/eLife.32273)
Supplement: Figure 6—source data 1. [file elife-32273-fig6-data1.docx]

**Figure 6-source data 1. The DNA and translated protein sequences for the *bab* open reading frames.**

***D. melanogaster bab1* ORF**

gaattcaacttaaaaaaaaaaatcaaaatggcgtcggcgcaggcggagacgaatgtcggc

M A S A Q A E T N V G

ttggcgtccgaacagggaccagtggctcagaggcagcgcaaagggacgggatcgggcgcc

L A S E Q G P V A Q R Q R K G T G S G A

gattcgcccaagagtaacagaagctcgcccactcagcaggaggagaagcgtatcaaaagc

D S P K S N R S S P T Q Q E E K R I K S

gaggatcgcacttcaccaactggcggggccaaggacgaggacaaggagagtcaaggtcat

E D R T S P T G G A K D E D K E S Q G H

gctgtagccggagggggaggatcttcgcccgtcagttcgccacagggcaggagttcttcg

A V A G G G G S S P V S S P Q G R S S S

gtagcctcgcccagttccagctcccagcaattctgcctgcgctggaacaactatcagacg

V A S P S S S S Q Q F C L R W N N Y Q T

aacctgaccaccatctttgaccagctgctccagaacgagtgcttcgtggacgtgaccttg

N L T T I F D Q L L Q N E C F V D V T L

gcatgcgatggtcggtccatgaaggcccacaagatggtcctgtccgcctgctcgccctac

A C D G R S M K A H K M V L S A C S P Y

ttccaaacacttctggccgaaacgccctgccagcatcccattgtgatcatgcgggacgta

F Q T L L A E T P C Q H P I V I M R D V

aattggtcggatctcaaggccattgtggagttcatgtatcgcggcgagatcaacgtgagc

N W S D L K A I V E F M Y R G E I N V S

caggaccagataggtcctctgctcaggatagctgagatgttgaaagtgcgtggtctggcg

Q D Q I G P L L R I A E M L K V R G L A

gatgtgacccatatggaggcggccacggcagcagcggctgccgcttcgtcggagagaatg

D V T H M E A A T A A A A A A S S E R M

ccctcctcgcccaaggagagcacttcaacttccagaactgaacacgacagggaacgggag

P S S P K E S T S T S R T E H D R E R E

gccgaggagctgctggccttcatgcagcccgagaagaagctacgcacttcggactgggac

A E E L L A F M Q P E K K L R T S D W D

cccgctgagctgaggctctccccactggagcggcagcagggcaggaatgtaagaaagcgc

P A E L R L S P L E R Q Q G R N V R K R

cggtggccatcggcggacacaatattcaatccacccgcaccacccagtccactgagcagc

R W P S A D T I F N P P A P P S P L S S

ctgattgcggccgaaaggatggagctggagcaaaaggaaagagagagacagagggactgt

L I A A E R M E L E Q K E R E R Q R D C

tcgctgatgacacccccacccaaaccaccaatgagcagtggctccacagtgggagccacg

S L M T P P P K P P M S S G S T V G A T

aggcgcctggagaccgccatccacgccctggacatgccatcgccggctgccacgccagga

R R L E T A I H A L D M P S P A A T P G

cctctgtcccgatcgtcgagacctcactcgcagagcccccagcagcagcaggcacagcag

P L S R S S R P H S Q S P Q Q Q Q A Q Q

cagggtcagcttcctttgcccctgcccctgcatccgcaccatcacgcatcacccgcccca

Q G Q L P L P L P L H P H H H A S P A P

catccctcccagaccgccggatcagcccaccacccggcatcgcctgctggagattcccgt

H P S Q T A G S A H H P A S P A G D S R

tttcccctcggcccagcagccgccatggccgctgccagggaactgagtggcctgggacca

F P L G P A A A M A A A R E L S G L G P

ggtccgtccgccgagccacgccttccgcctccaccgccgcaccaccatggcggtggtgga

G P S A E P R L P P P P P H H H G G G G

gtgggcggcgggggagttggaggaggaggtgcaggcggagtgggttcaggcgggggatcc

V G G G G V G G G G A G G V G S G G G S

tcgctcgccgatgacttggagatcaagccagggatcgccgagatgatccgagaggaagaa

S L A D D L E I K P G I A E M I R E E E

agggccaaaatgatggagaactcgcacgcctggatgggcgccaccggatcaacgctggca

R A K M M E N S H A W M G A T G S T L A

gcagacagctaccagtaccagctgcagtccatgtggcaaaagtgctggaacaccaaccag

A D S Y Q Y Q L Q S M W Q K C W N T N Q

aatctgatgcatcacatgcgcttccgcgagcgaggtcctctgaagtcgtggcgacccgag

N L M H H M R F R E R G P L K S W R P E

accatggcggaggccattttcagtgtgctaaaggagggtctatcgctatctcaggccgcc

T M A E A I F S V L K E G L S L S Q A A

cgcaagtacgacatcccgtatccaacattcgtgctctatgcgaacagggtgcacaatatg

R K Y D I P Y P T F V L Y A N R V H N M

ctgggaccatccattgacggcgggcccgatttgcggcccaaggggcgtggcaggccgcag

L G P S I D G G P D L R P K G R G R P Q

cgaatccttctgggcatctggcccgacgagcacattaagggcgtcatcaagacggtggtc

R I L L G I W P D E H I K G V I K T V V

tttcgcgacaccaaggacatcaaggacgagagcctggccgctcacatgccaccctacggt

F R D T K D I K D E S L A A H M P P Y G

cgacattcgcccgcgtttcccttgcaggacctccctctcagctatcccggagccagtggc

R H S P A F P L Q D L P L S Y P G A S G

gccctggcaggcgcgcccagctccatggcctgtccgaatggcagtggaccgcagaccgga

A L A G A P S S M A C P N G S G P Q T G

gtgggcgtggccggagagcagcatatgtcacaggaaacggccgccgcggtggccgccgtg

V G V A G E Q H M S Q E T A A A V A A V

gcgcacaacatccgccagcagatgcaaatggcagcggttccgcccggcttattcaatctg

A H N I R Q Q M Q M A A V P P G L F N L

ccgcctcatccgggagtgggcggtggagtgggcaacgttcccggcgcagctggaggcagg

P P H P G V G G G V G N V P G A A G G R

gccagcatatcgccggccctgagcagtggctccggaccaaggcacgctccctcgccctgc

A S I S P A L S S G S G P R H A P S P C

ggtcccgccggcctcctgccgaacctgccgcccagcatggccgtcgctctgcaccaccag

G P A G L L P N L P P S M A V A L H H Q

cagcaacagcaggcggcgcaccaccacatgcagcagctgcacctgcagcagcaacaggcc

Q Q Q Q A A H H H M Q Q L H L Q Q Q Q A

cacttgcaccaccatcagcagcaacagcaacagcagcagcagcagcaccatcagggcggc

H L H H H Q Q Q Q Q Q Q Q Q Q H H Q G G

catcaggtggcccacaagtccggtttcggtgccagctccagttcctcagcctcctcgtcg

H Q V A H K S G F G A S S S S S A S S S

tcaatgggccagcaccatgcgcccaaggccaagagcagtccgttgcgcagcgaaacgcct

S M G Q H H A P K A K S S P L R S E T P

cggctgcactccccgctcggcgatcttggcctggacatggccagctacaagcgcgagttc

R L H S P L G D L G L D M A S Y K R E F

tcgcccagccgcctcttcgccgaggatctggccgagctggtgggcgccagtgtctcatct

S P S R L F A E D L A E L V G A S V S S

tcctcatcatcggcggcggcagcgacggctcctccggaaagatcggcaggagcagcttcc

S S S S A A A A T A P P E R S A G A A S

gcagccacaggcgcggatgcacccagttcctcgagcagtggaggcatcaaggtggaaccc

A A T G A D A P S S S S S G G I K V E P

Attaccaccactagcgagtaataggcggccgc

I T T T S E - - - -

***D. melanogaster bab1* DNA-binding mutant ORF**

gaattcaacttaaaaaaaaaaatcaaaatggcgtcggcgcaggcggagacgaatgtcggc

M A S A Q A E T N V G

ttggcgtccgaacagggaccagtggctcagaggcagcgcaaagggacgggatcgggcgcc

L A S E Q G P V A Q R Q R K G T G S G A

gattcgcccaagagtaacagaagctcgcccactcagcaggaggagaagcgtatcaaaagc

D S P K S N R S S P T Q Q E E K R I K S

gaggatcgcacttcaccaactggcggggccaaggacgaggacaaggagagtcaaggtcat

E D R T S P T G G A K D E D K E S Q G H

gctgtagccggagggggaggatcttcgcccgtcagttcgccacagggcaggagttcttcg

A V A G G G G S S P V S S P Q G R S S S

gtagcctcgcccagttccagctcccagcaattctgcctgcgctggaacaactatcagacg

V A S P S S S S Q Q F C L R W N N Y Q T

aacctgaccaccatctttgaccagctgctccagaacgagtgcttcgtggacgtgaccttg

N L T T I F D Q L L Q N E C F V D V T L

gcatgcgatggtcggtccatgaaggcccacaagatggtcctgtccgcctgctcgccctac

A C D G R S M K A H K M V L S A C S P Y

ttccaaacacttctggccgaaacgccctgccagcatcccattgtgatcatgcgggacgta

F Q T L L A E T P C Q H P I V I M R D V

aattggtcggatctcaaggccattgtggagttcatgtatcgcggcgagatcaacgtgagc

N W S D L K A I V E F M Y R G E I N V S

caggaccagataggtcctctgctcaggatagctgagatgttgaaagtgcgtggtctggcg

Q D Q I G P L L R I A E M L K V R G L A

gatgtgacccatatggaggcggccacggcagcagcggctgccgcttcgtcggagagaatg

D V T H M E A A T A A A A A A S S E R M

ccctcctcgcccaaggagagcacttcaacttccagaactgaacacgacagggaacgggag

P S S P K E S T S T S R T E H D R E R E

gccgaggagctgctggccttcatgcagcccgagaagaagctacgcacttcggactgggac

A E E L L A F M Q P E K K L R T S D W D

cccgctgagctgaggctctccccactggagcggcagcagggcaggaatgtaagaaagcgc

P A E L R L S P L E R Q Q G R N V R K R

cggtggccatcggcggacacaatattcaatccacccgcaccacccagtccactgagcagc

R W P S A D T I F N P P A P P S P L S S

ctgattgcggccgaaaggatggagctggagcaaaaggaaagagagagacagagggactgt

L I A A E R M E L E Q K E R E R Q R D C

tcgctgatgacacccccacccaaaccaccaatgagcagtggctccacagtgggagccacg

S L M T P P P K P P M S S G S T V G A T

aggcgcctggagaccgccatccacgccctggacatgccatcgccggctgccacgccagga

R R L E T A I H A L D M P S P A A T P G

cctctgtcccgatcgtcgagacctcactcgcagagcccccagcagcagcaggcacagcag

P L S R S S R P H S Q S P Q Q Q Q A Q Q

cagggtcagcttcctttgcccctgcccctgcatccgcaccatcacgcatcacccgcccca

Q G Q L P L P L P L H P H H H A S P A P

catccctcccagaccgccggatcagcccaccacccggcatcgcctgctggagattcccgt

H P S Q T A G S A H H P A S P A G D S R

tttcccctcggcccagcagccgccatggccgctgccagggaactgagtggcctgggacca

F P L G P A A A M A A A R E L S G L G P

ggtccgtccgccgagccacgccttccgcctccaccgccgcaccaccatggcggtggtgga

G P S A E P R L P P P P P H H H G G G G

gtgggcggcgggggagttggaggaggaggtgcaggcggagtgggttcaggcgggggatcc

V G G G G V G G G G A G G V G S G G G S

tcgctcgccgatgacttggagatcaagccagggatcgccgagatgatccgagaggaagaa

S L A D D L E I K P G I A E M I R E E E

agggccaaaatgatggagaactcgcacgcctggatgggcgccaccggatcaacgctggca

R A K M M E N S H A W M G A T G S T L A

gcagacagctaccagtaccagctgcagtccatgtggcaaaagtgctggaacaccaaccag

A D S Y Q Y Q L Q S M W Q K C W N T N Q

aatctgatgcatcacatgcgcttccgcgagcgaggtcctctgaagtcgtggcgacccgag

N L M H H M R F R E R G P L K S W R P E

accatggcggag**gGcCCt**ttcagtgtgctaaaggagggtctatcgctatctcaggccgcc

T M A E G P F S V L K E G L S L S Q A A

cgcaagtacgacatcccgtatccaacattcgtgctctatgcgaacagggtgcacaatatg

R K Y D I P Y P T F V L Y A N R V H N M

ctgggaccatccattgacggcgggcccgatttgcggcccaagggg**GAtggcGA**Cccgcag

L G P S I D G G P D L R P K G D G D P Q

cgaatccttctgggcatctggcccgacgagcacattaagggcgtcatcaagacggtggtc

R I L L G I W P D E H I K G V I K T V V

tttcgcgacaccaaggacatcaaggacgagagcctggccgctcacatgccaccctacggt

F R D T K D I K D E S L A A H M P P Y G

cgacattcgcccgcgtttcccttgcaggacctccctctcagctatcccggagccagtggc

R H S P A F P L Q D L P L S Y P G A S G

gccctggcaggcgcgcccagctccatggcctgtccgaatggcagtggaccgcagaccgga

A L A G A P S S M A C P N G S G P Q T G

gtgggcgtggccggagagcagcatatgtcacaggaaacggccgccgcggtggccgccgtg

V G V A G E Q H M S Q E T A A A V A A V

gcgcacaacatccgccagcagatgcaaatggcagcggttccgcccggcttattcaatctg

A H N I R Q Q M Q M A A V P P G L F N L

ccgcctcatccgggagtgggcggtggagtgggcaacgttcccggcgcagctggaggcagg

P P H P G V G G G V G N V P G A A G G R

gccagcatatcgccggccctgagcagtggctccggaccaaggcacgctccctcgccctgc

A S I S P A L S S G S G P R H A P S P C

ggtcccgccggcctcctgccgaacctgccgcccagcatggccgtcgctctgcaccaccag

G P A G L L P N L P P S M A V A L H H Q

cagcaacagcaggcggcgcaccaccacatgcagcagctgcacctgcagcagcaacaggcc

Q Q Q Q A A H H H M Q Q L H L Q Q Q Q A

cacttgcaccaccatcagcagcaacagcaacagcagcagcagcagcaccatcagggcggc

H L H H H Q Q Q Q Q Q Q Q Q Q H H Q G G

catcaggtggcccacaagtccggtttcggtgccagctccagttcctcagcctcctcgtcg

H Q V A H K S G F G A S S S S S A S S S

tcaatgggccagcaccatgcgcccaaggccaagagcagtccgttgcgcagcgaaacgcct

S M G Q H H A P K A K S S P L R S E T P

cggctgcactccccgctcggcgatcttggcctggacatggccagctacaagcgcgagttc

R L H S P L G D L G L D M A S Y K R E F

tcgcccagccgcctcttcgccgaggatctggccgagctggtgggcgccagtgtctcatct

S P S R L F A E D L A E L V G A S V S S

tcctcatcatcggcggcggcagcgacggctcctccggaaagatcggcaggagcagcttcc

S S S S A A A A T A P P E R S A G A A S

gcagccacaggcgcggatgcacccagttcctcgagcagtggaggcatcaaggtggaaccc

A A T G A D A P S S S S S G G I K V E P

attaccaccactagcgagtaataggcggccgc

I T T T S E - - - -

***D. melanogaster bab2* ORF**

gaattcaacttaaaaaaaaaaatcaaaatggacatgaccaaacagattgtggactttgaa

M D M T K Q I V D F E

ataaagtcggaactgatcggcgaaatcgatcagttcgaggcgagtgactacacaatggct

I K S E L I G E I D Q F E A S D Y T M A

ccaccggaagagcctaagatggtggaagagtccccccagttgggtcatctagaggaccag

P P E E P K M V E E S P Q L G H L E D Q

aacagaaagtattcacccgaaagggaggttgaacccactctgcaggatccaagtgaggtg

N R K Y S P E R E V E P T L Q D P S E V

gttgatcaaatgcaaaaagatacggagagcgttggagaagtcaagtcacccgagaaggat

V D Q M Q K D T E S V G E V K S P E K D

gtggaaacggagctggtgaagtccaaggcgagtccgatgaacgaccaagctttgactccc

V E T E L V K S K A S P M N D Q A L T P

ccaccacgacctctgacctccagtgaagtggtgggtctccgggatcccgaacataccgag

P P R P L T S S E V V G L R D P E H T E

ctgcgcatgtgcctggaggccaagaagtcgcgctccctaccagtttccccacagcctcaa

L R M C L E A K K S R S L P V S P Q P Q

ccaaatcttaagctagccggatcggcgctctttgagttcggtcagagatcctctcccgtg

P N L K L A G S A L F E F G Q R S S P V

gagaccaagatcaaaaccaatccagagacaaaaccgccgaggcgcaaaatagttcctccc

E T K I K T N P E T K P P R R K I V P P

agcggcgaggggcagcagttctgcctgaggtggaacaactatcagtctaacctgaccaat

S G E G Q Q F C L R W N N Y Q S N L T N

gtctttgacgaactccttcagagcgagtccttcgtggacgtgaccttgtcctgcgaaggc

V F D E L L Q S E S F V D V T L S C E G

cactcgatcaaggcacacaagatggtgctatccgcctgctcaccctacttccaggccctg

H S I K A H K M V L S A C S P Y F Q A L

ttctacgacaatccctgccagcaccccatcatcatcatgcgggacgtcagctggtccgac

F Y D N P C Q H P I I I M R D V S W S D

ctgaaggccctggtggagttcatgtacaagggggagatcaacgtctgccaggatcagata

L K A L V E F M Y K G E I N V C Q D Q I

aaccccctgctcaaagtggccgaaaccctgaagatcaggggtctggcggaggtcagtgcg

N P L L K V A E T L K I R G L A E V S A

ggcaggggcgagggaggcgcctccgcacttcccatgtccgccttcgacgatgaggacgag

G R G E G G A S A L P M S A F D D E D E

gaggaggaactggcctcggccactgcaattctgcagcaggacggtgatgccgatcccgat

E E E L A S A T A I L Q Q D G D A D P D

gaggagatgaaggccaagaggcccagactgctgcccgagggagtcttggacttgaatcag

E E M K A K R P R L L P E G V L D L N Q

cgacaaaggaagcggtccagggatggcagctacgccactccaagtccatcccttcagggc

R Q R K R S R D G S Y A T P S P S L Q G

ggagagtccgagatctcggagaggggctcatccggcactccgggacagagccagagccaa

G E S E I S E R G S S G T P G Q S Q S Q

cccctggccatgaccacctccaccattgtgcgcaatcccttcgcctcccccaatcctcag

P L A M T T S T I V R N P F A S P N P Q

accttggagggcaggaacagcgccatgaatgcagtagcaaaccagaggaaatcaccagca

T L E G R N S A M N A V A N Q R K S P A

ccaacagcgacaggtcacagcaatgggaacagcggcgccgccatgcactccccacccggg

P T A T G H S N G N S G A A M H S P P G

ggcgtggccgtccagtccgcccttccgccccacatggccgccatcgtgccgccacccccc

G V A V Q S A L P P H M A A I V P P P P

tccgccatgcaccatcatgcccagcaactggccgcccagcaccagctggcccactcgcac

S A M H H H A Q Q L A A Q H Q L A H S H

gccatggccagcgccttggcagccgcagccgccggagctggcgcagcgggagcgggcgga

A M A S A L A A A A A G A G A A G A G G

gcaggatctggcagtggatcgggcgccagtgctccgactggaggaacaggagtggcggga

A G S G S G S G A S A P T G G T G V A G

agtggagccggcgcggcggtgggatcccatcacgatgacatggagatcaagccagaaatc

S G A G A A V G S H H D D M E I K P E I

gccgagatgatacgcgaagaagagagggccaagatgatcgagagtggaggccacggtgga

A E M I R E E E R A K M I E S G G H G G

tggatgggagcggcagctgcggcaactggagcagcttctgtggcggcagatagctaccag

W M G A A A A A T G A A S V A A D S Y Q

taccagctacagtccatgtggcagaagtgctggaacaccaatcagcagaacctggtgcag

Y Q L Q S M W Q K C W N T N Q Q N L V Q

cagctcagattccgcgagcgcggcccattgaagtcctggcgacccgaggccatggccgag

Q L R F R E R G P L K S W R P E A M A E

gccattttcagtgtcctgaaggaggggctctccctgtcacaggctgcccgcaagttcgac

A I F S V L K E G L S L S Q A A R K F D

ataccctatcccaccttcgtcctgtacgccaatcgggtgcacaacatgctgggaccctcg

I P Y P T F V L Y A N R V H N M L G P S

ctggatggcggagctgatccgcggccaaaggcacgcggtcgtccccagaggatcctgctg

L D G G A D P R P K A R G R P Q R I L L

ggcatgtggccggaggagctcatccgtagcgtcattaaggccgtggtgttccgggactat

G M W P E E L I R S V I K A V V F R D Y

cgcgagattaaggaggacatgagcgcccatcagtacgccaatggacagggtcatggtacc

R E I K E D M S A H Q Y A N G Q G H G T

tatatcggaggaggaaccaccacgaatggctaccacagtgctgccgcagccaagctggcg

Y I G G G T T T N G Y H S A A A A K L A

gctcagaacgctgcactggctccgccggacgcaggaagtccgctgagctccatgacggaa

A Q N A A L A P P D A G S P L S S M T E

acccttcgccgccagatcctctcgcagcagcagcaacatcagcagcaccaccagcagcag

T L R R Q I L S Q Q Q Q H Q Q H H Q Q Q

gcacaccatcagcaacagccctcgcaccaccagcaacagtcgccccacgcccagtccatg

A H H Q Q Q P S H H Q Q Q S P H A Q S M

aacatgtacaagtccccggcctatctgcagcgatccgagatcgaagatcaagtatccgca

N M Y K S P A Y L Q R S E I E D Q V S A

gcggcggccgtggcagcggcggcggccaagcaccagcagcagcagggtgagcgaaggggt

A A A V A A A A A K H Q Q Q Q G E R R G

tcggagaacctgcccgacctcagtgccctgggcctgatgggtctgcccggcctgaatgtg

S E N L P D L S A L G L M G L P G L N V

atgccctcacggggatcgggtggaggaagtggtggcgcagcgccgaatagtgccgcctcc

M P S R G S G G G S G G A A P N S A A S

tatgcccgcgagttatcccgcgaaagggaacgcgatcgggagcgcgaaagggagcgggag

Y A R E L S R E R E R D R E R E R E R E

ctgtcccgccagtatggcagccagtcgcggggatcgagctccggttccggaagcgccaag

L S R Q Y G S Q S R G S S S G S G S A K

tccctgaccgccagccaaagaccaggagccgcctcgccgtactccgccgcccactatgcc

S L T A S Q R P G A A S P Y S A A H Y A

aaacatcaggcgagtgcctacaacaagaggtttctcgagagcctgcccgccggcattgac

K H Q A S A Y N K R F L E S L P A G I D

ttggaggccttcgccaacggactgctccagaagtcggtgaacaagagtccgcgcttcgag

L E A F A N G L L Q K S V N K S P R F E

gacttcttcccgggacccggccaggacatgagtgaactgtttgccaatccggacgcgagt

D F F P G P G Q D M S E L F A N P D A S

gcagctgccgcggcggcggcctacgcgcctcctggcgccatccgcgaatcgcctctgatg

A A A A A A A Y A P P G A I R E S P L M

aagatcaagctggagcagcagcatgccaccgaactgccgcacgaggattgataggcggcc

K I K L E Q Q H A T E L P H E D - - - -

Gc

***A. gambiae bab* ORF**

gaattcaacttaaaaaaaaaaatcaaaatgggcaagccaatcccgaaccccctgctgggc

M **G K P I P N P L L G**

ctggactccacaccaagcgatacacccccgccgagcgccacctccgtgagccacccatcg

**L D S T** P S D T P P P S A T S V S H P S

cccgccagttcgcaccatgatcccaacgatccaaatgcccccccgcgcgatcccgtggat

P A S S H H D P N D P N A P P R D P V D

cgcagtggcacaggaaccccgggccccagcgatcacccaacaggcggacacctgggccac

R S G T G T P G P S D H P T G G H L G H

catcagccaccgtcctcgtcgagtagctcctcgagctcgagttccagctcgtcgacaagc

H Q P P S S S S S S S S S S S S S S T S

tcctcgctgtcctcgctgagtctgaagcgctccctggaggagcccctgacgaccgccaag

S S L S S L S L K R S L E E P L T T A K

ccatcgccgccctgcagccccctgaccatggaccaccatcatcagcataaggccgcccgc

P S P P C S P L T M D H H H Q H K A A R

cagtcgcgcgccgcctcgcccgccggacgcagtacgcagcagcaggcctcgccaagtgcc

Q S R A A S P A G R S T Q Q Q A S P S A

cccggcaccggcggatcgagtagcggaggcggcggaggaggccagcagttctgcctgcgc

P G T G G S S S G G G G G G Q Q F C L R

tggaataactaccagaccaatctgaccagcgtgttcgaccagctgctgcagagcgagtcg

W N N Y Q T N L T S V F D Q L L Q S E S

ttcgtggatgtgaccctggcctgcgatggccagagcatgaaggcccataagatggtgctg

F V D V T L A C D G Q S M K A H K M V L

agcgcctgctcgccgtacttccagacgctgtttttcgataacccgtgccagcatcccatt

S A C S P Y F Q T L F F D N P C Q H P I

gtgatcatgcgcgacgtgtcgtgggccgagctgaaggccatcgtggagttcatgtacaag

V I M R D V S W A E L K A I V E F M Y K

ggcgagatcaatgtgtcgcaggatcagatcggccccctgctgaaggtggccgagatgctg

G E I N V S Q D Q I G P L L K V A E M L

aagatccgcggcctggccgatgtgagcggagacgccggagagccaacaggaagccgcgcc

K I R G L A D V S G D A G E P T G S R A

gagcgcgaggccgccggaagccgcggccccgaggagctggatcgcgaggagcatggcaag

E R E A A G S R G P E E L D R E E H G K

ctgctgaaccccctggccatcgtgggatcgagcctgctggccaatggagccgccagcgcc

L L N P L A I V G S S L L A N G A A S A

gccatggccggaggcaacggcagtaacagcaccgccacaagcggctccgccgccgtgcag

A M A G G N G S N S T A T S G S A A V Q

gccgccgccgccgccgccgccgccaagaagcagcgcgccggacgcgatcgcgacacaacc

A A A A A A A A K K Q R A G R D R D T T

aaggagcaccgcatggatgcccgcctgtcggagtttgcccgcgacctgagccgcgccgat

K E H R M D A R L S E F A R D L S R A D

ccccacatctcgagccgcgatatcagcagtgtggccgccgccgccgccgccgccgccgcc

P H I S S R D I S S V A A A A A A A A A

gccgccgccggcctggccgtgggagagtggcccctgggagccgccggcctggaggccgcc

A A A G L A V G E W P L G A A G L E A A

gccgccgccgccgtgcaggcctcgacacccaagtccgcccgcaagcgccgctggccctcg

A A A A V Q A S T P K S A R K R R W P S

ggagagcgctcgagcattggatcgccagccgacagcaccccggaccagctggaggtgcca

G E R S S I G S P A D S T P D Q L E V P

tcgccgatcccacccacaccgagtagcctggcccagtcgagcggaggaggcggaggcggc

S P I P P T P S S L A Q S S G G G G G G

ggcggcggaggcggaggcggcacaggaagtggcggcggcggaggcggcagctcgaatccg

G G G G G G G T G S G G G G G G S S N P

ctggcctcctttccgctgccacccgccctggacaccgccgccatggccatgagtagcctg

L A S F P L P P A L D T A A M A M S S L

tccagttcgatcgccaatcacccagacgacatggagatcaagccgggaattgccgagatg

S S S I A N H P D D M E I K P G I A E M

atccgcgaggaggagcgcagcatgtggcagaagtgctggaacagccagaacctgatccac

I R E E E R S M W Q K C W N S Q N L I H

catctgcgctttcgcgagcgcggacccctgaagtcgtggcgcccggagacaatggccgag

H L R F R E R G P L K S W R P E T M A E

gccatcttcagcgtgctgaaggagggactgtcgctgagccaggccgcccgcaagtatgac

A I F S V L K E G L S L S Q A A R K Y D

atcccatatcccaccttcgtgctgtacgccaaccgcgtgcataacatgctgggccccagc

I P Y P T F V L Y A N R V H N M L G P S

atcgatggaggcaccgacctgcgccccaagggccgcggacgcccccagcgcattctgctg

I D G G T D L R P K G R G R P Q R I L L

ggcatctggcccgacgatcacatcaagggagtgatcaagtccgtggtgttccgcgatgcc

G I W P D D H I K G V I K S V V F R D A

aaggacatgaaggaggagccgatgatgtatggacgccacagtccgttccccttccaggat

K D M K E E P M M Y G R H S P F P F Q D

aacccgctgagctacggaccaaccgccccaaatggccagctgccctcggtggccacaggc

N P L S Y G P T A P N G Q L P S V A T G

accaacgtgcccgatggcatgagccaggacgccctgaccgccgccacagtggccgccgtg

T N V P D G M S Q D A L T A A T V A A V

cgccagcagatgtgcaacatggtggccgccgcccagcaccacccagatgccgccaacctg

R Q Q M C N M V A A A Q H H P D A A N L

gtggccgccgccggatttaacctgccatcccactgcggcacccccccgaatctgtcgatg

V A A A G F N L P S H C G T P P N L S M

cacccagccgccgccgccgccgccgccgccgccgccgccgcctcgaatgcctcggccgcc

H P A A A A A A A A A A A A S N A S A A

ggaggcccgtccggaggaggaggcggaggaggctcgagcggcgccattccgctgccgaag

G G P S G G G G G G G S S G A I P L P K

atgggatcgccagccgtgccgtccacgggccacggaaacaataacggcggaagtggcgcc

M G S P A V P S T G H G N N N G G S G A

ggcattcagatgccacgcctgggaagccccgccggatcgagcggcctggccaaggagcat

G I Q M P R L G S P A G S S G L A K E H

gagctgcagcatcacggcggaggaggaggaggaggcggcggcctgggaggcggatcgggc

E L Q H H G G G G G G G G G L G G G S G

ggaggaatgagccgcgccaccccccccggcgcccgcgatcgcgccatgaccgcccgctcc

G G M S R A T P P G A R D R A M T A R S

aatctggccgccggagagaccggacgcagcagttcgagcgccggctcgatccaccgctcc

N L A A G E T G R S S S S A G S I H R S

agcccgagctcctcggccggctcgagtctgaatcaccagcatcccgcccacctgtcccac

S P S S S A G S S L N H Q H P A H L S H

ccacaccatcagcagcagcatcaccatcagcatcatcaccagccgcaccatggacatgcc

P H H Q Q Q H H H Q H H H Q P H H G H A

catcacctgccacatcacaaccccctgagtcatctggtgggatcgggaggagccagcggc

H H L P H H N P L S H L V G S G G A S G

gccctgagcatcaccaagctgggctcccccggaagtgcccacgatctgcgcatctcgaat

A L S I T K L G S P G S A H D L R I S N

agtcccgacgagagtccactggccagcccaattggactggccatggagccagccgtgaat

S P D E S P L A S P I G L A M E P A V N

ctggccctgggcgccggaggaacccagcccggccccgaggatgtgcgcctgcatgtgccc

L A L G A G G T Q P G P E D V R L H V P

ccgccatacggctcgaagccgcccagccgcggcggaggagcccccagcaccggatacacg

P P Y G S K P P S R G G G A P S T G Y T

agcaactcgagtccgccccgcccagagcatctgttccaggatcaggacattgccgccctg

S N S S P P R P E H L F Q D Q D I A A L

gtggccaccacgcgcgccgcctgcccccccagccgcgtgcccgattacaaggacaccgcc

V A T T R A A C P P S R V P D Y K D T A

gtgcgcccgacagccagcatcaaggtggagcccctgacagagtgccgcggagactaatag

V R P T A S I K V E P L T E C R G D - -

gcggccgc

- -

***D. mojavensis bab2* ORF**

gaattcaacttaaaaaaaaaaatcaaaatggacatgacaaaggacattatggacttcgag

M D M T K D I M D F E

cgcaagagcctggatagttcgtgcggagagcagttcgagccgtcggattacacaatggtg

R K S L D S S C G E Q F E P S D Y T M V

aatgccgagctggccaagcaggccgcccagaccgcccaggccgtggatcaggtggagctg

N A E L A K Q A A Q T A Q A V D Q V E L

gacctgccactggagctggccaagaaggaggagccggaggcccagcccgagcccatgcag

D L P L E L A K K E E P E A Q P E P M Q

cagctgaaggaggagaatcgcgccgtggccgccgagaagcccgccatgctgaatgagcag

Q L K E E N R A V A A E K P A M L N E Q

gccctgacccccccaccgcgccccctgaccagcagcgaggtggtgggccactccgagcca

A L T P P P R P L T S S E V V G H S E P

tcggacccggagctgcagattcagctgaccgccaagaagtcgcgcagcctgccggtgtcg

S D P E L Q I Q L T A K K S R S L P V S

ccacagccactggtggcccataatctggccgccatcggactgtttgagttcggaaagacg

P Q P L V A H N L A A I G L F E F G K T

gtggagacacccgagctgaagcccaagatgaatcacaagctgctgcccccggtgaacgtg

V E T P E L K P K M N H K L L P P V N V

ggcgtggccccacgcaaggtggcccccagcgccggcggaggcgacaatcagcagttctgc

G V A P R K V A P S A G G G D N Q Q F C

ctgcgctggaataactaccagagcaacctgaccaacgtgttcgacgagctgctgcagaac

L R W N N Y Q S N L T N V F D E L L Q N

gagtcctttgtggacgtgaccctggcctgcgatggccagagcattaaggcccacaagatg

E S F V D V T L A C D G Q S I K A H K M

gtgctgtcggcctgctccccctacttccaggccctgttctacgataacccgtgccagcac

V L S A C S P Y F Q A L F Y D N P C Q H

cccattatcatcatgcgcgatgtgaactggtgcgacctgaaggccctggtggagttcatg

P I I I M R D V N W C D L K A L V E F M

tacaagggagagatcaacgtgtgccaggaccagattaatcccctgctgaaggtggccgag

Y K G E I N V C Q D Q I N P L L K V A E

accctgaagattcgcggcctggccgaggtgggcgcctcgtccaccgccgccggcctgggc

T L K I R G L A E V G A S S T A A G L G

gccgccagcatgctgcccgagcagcgcatgagcgtgtatgacgatgaggaggatgaggac

A A S M L P E Q R M S V Y D D E E D E D

gagctggccgccgccgccgccctgctgaacgatgaggatgaggatgagctgctgaagcca

E L A A A A A L L N D E D E D E L L K P

aagcgcgcccgcctgctggccaagctgcgcgccgccgagaccgccctggatctgaaccag

K R A R L L A K L R A A E T A L D L N Q

cgccagcgcaagcgctcccgcgatggcagctacgccaccccctcgccactgcgcagcgag

R Q R K R S R D G S Y A T P S P L R S E

tcgccgagttcgcagctgccactggccatgacgaccagcaccattgtgcgcaatcccttt

S P S S Q L P L A M T T S T I V R N P F

gccagccccaatccccagaccctgccagcctcgagtggaagttcgtccaacagtaacagc

A S P N P Q T L P A S S G S S S N S N S

aataacagctcgtgcaacaactcgtccagcaacagttccagcaccgccacagccgccgcc

N N S S C N N S S S N S S S T A T A A A

cagccgaccgccacaaactgcagcagctccagtagcgccggcgtgccaagcaacggaagt

Q P T A T N C S S S S S A G V P S N G S

agctcggccgcctatcgcagtccacccccaccgcccccccccccgtcgtccgcccatagc

S S A A Y R S P P P P P P P P S S A H S

aatggatcgagcgccgccggactgagctcgcccacaggaaacaagagctccgccgccgcc

N G S S A A G L S S P T G N K S S A A A

gccgccgcccagagccagctgccaccccatatggccgccgccgtggccgccgccgcccat

A A A Q S Q L P P H M A A A V A A A A H

cacgcctccgccaatgtgccgcccccaccaccaggagccgccgcctcgatgcaccatcac

H A S A N V P P P P P G A A A S M H H H

gccgccgccgccgccgcccagcagctggccgcccagcaccagctggcccacagccatgcc

A A A A A A Q Q L A A Q H Q L A H S H A

gccatggccagcgtgctgggcgcctcgctggccgccgccgccgccggaggcgccgccgcc

A M A S V L G A S L A A A A A G G A A A

cccggctccgccgccggcgccggaaatgccccaagctcggtgggaggacaccatgacgat

P G S A A G A G N A P S S V G G H H D D

atggagatcaagcccgagattgccgagatgattcgcgaggaggagcgcgccaagatgatc

M E I K P E I A E M I R E E E R A K M I

gagaccagcggccatgcctggatgggcgccccagccacaggagcctcggtggccgccgac

E T S G H A W M G A P A T G A S V A A D

agctaccagtaccagctgcagagcatgtggcagaagtgctggaataccaaccagcagaac

S Y Q Y Q L Q S M W Q K C W N T N Q Q N

ctggtgcagcagctgcgctttcgcgagcgcggaccactgaagtcgtggcgccccgaggcc

L V Q Q L R F R E R G P L K S W R P E A

atggccgaggccatcttttcggtgctgaaggagggactgagtctgagccaggccgcccgc

M A E A I F S V L K E G L S L S Q A A R

aagtacgacatcccataccccacctttgtgctgtatgccaaccgcgtgcataatatgctg

K Y D I P Y P T F V L Y A N R V H N M L

ggcccaagcctggatggaggatcggatccacgccccaaggcccgcggacgcccccagcgc

G P S L D G G S D P R P K A R G R P Q R

atcctgctgggaatgtggccagatgagctgattcgctcggtgatcaaggccgtggtgttc

I L L G M W P D E L I R S V I K A V V F

cgcgattaccgcgagattaaggaggatatcaacgcccatccctatgccaacggccagccc

R D Y R E I K E D I N A H P Y A N G Q P

cacggagcccattacggctccaatagtgccgccgccgccaacggataccacagcgccaca

H G A H Y G S N S A A A A N G Y H S A T

gtgaagatggccccgcccgatgccagcaacccgctgagtaccatgacggagaccctgcgc

V K M A P P D A S N P L S T M T E T L R

cgccagatcctgagccagcagcagcagcagcagcagcagcagcagcagcagcagcaccag

R Q I L S Q Q Q Q Q Q Q Q Q Q Q Q Q H Q

cagcatcagcagcagagtccgcatatgcagtcgatgaatatgtacaagagtccagcctat

Q H Q Q Q S P H M Q S M N M Y K S P A Y

ctgcagcgctcggagattgaggaccaggtgtcggccgccgccgccgtggccgccgccaag

L Q R S E I E D Q V S A A A A V A A A K

catcagcagaatgagcgccgcggcagcgagaatctgccggatctgtcggccctgggactg

H Q Q N E R R G S E N L P D L S A L G L

atcggcctgcccggactgaatgtgatgccgacccagcagcagcccggcggacaccagcgc

I G L P G L N V M P T Q Q Q P G G H Q R

ggaggccccggctcgggcggagccgccgccggcggactgcatcccaacgccgccagctat

G G P G S G G A A A G G L H P N A A S Y

gcccgcgagctgagccgcgagcgcgagcgcgagcgcgagcgcgagcgcgagatgtcccgc

A R E L S R E R E R E R E R E R E M S R

gatcgcgagctgaaggaggccatgcacgcccgccagtacggaaaccagtcgcgcggctcc

D R E L K E A M H A R Q Y G N Q S R G S

aatagctccgccggcagcaagagtgccgcctcgagccgccccggcgccgccgccagcccg

N S S A G S K S A A S S R P G A A A S P

tactcggcccactacgccaagcatgccaaggagcacccatcgtacgcctataacaagcgc

Y S A H Y A K H A K E H P S Y A Y N K R

ttcctggagtcgctgcccgccggaatcgacttcgaggccattgccaacggcctgctgcag

F L E S L P A G I D F E A I A N G L L Q

aagagcgtgaataagtccccgcgctttgaggacttcttccccggacaggatatgagtgag

K S V N K S P R F E D F F P G Q D M S E

ctgtttggctccgccgatgccagtgccggatccggcgccggcagtgccgccgccgccgcc

L F G S A D A S A G S G A G S A A A A A

gccgccgccgccgccgccgccgcctacgccccacccggcatgcgcgagtcgccactgatg

A A A A A A A A Y A P P G M R E S P L M

aagattaagctggagcagcagcaggccgccgagctgccacacgaggattagtgagcggcc

K I K L E Q Q Q A A E L P H E D - - - -

gc

***D. willistoni bab1* ORF**

gaattcaacttaaaaaaaaaaatcaaaatggccaccctggagagcacgagtcagcgcaac

M A T L E S T S Q R N

gaccgcagcgagaccgagacgggaaatgagacaaatgtggagcaggcccagagcgcccag

D R S E T E T G N E T N V E Q A Q S A Q

cgccagcgcagtggaggcggcggatccaacggcggaggaggaggaggcggaattacgccc

R Q R S G G G G S N G G G G G G G I T P

accaagagccagcccgattccccgagcaacaagaccgaggatcagaagagcgagtcgacg

T K S Q P D S P S N K T E D Q K S E S T

cccgagcagcgccgcagcccaggaggcgtgcgcggagccgatggagccggctcgagtccc

P E Q R R S P G G V R G A D G A G S S P

gtggcctccccgcccgcccgcagcagttcggccgccagtcccaatagcaactccgcccag

V A S P P A R S S S A A S P N S N S A Q

cagttctgcctgcgctggaacaattaccagacaaatctgaccacaatcttcgatcagctg

Q F C L R W N N Y Q T N L T T I F D Q L

ctgcagaacgagtgcttcgtggacgtgacgctggcctgcgacggccgctccctgaaggcc

L Q N E C F V D V T L A C D G R S L K A

cacaagatggtgctgtcggcctgcagcccgtacttccagaccctgctggccgagacccca

H K M V L S A C S P Y F Q T L L A E T P

tgccagcatcccatcgtgattatgcgcgatgtgaactggtgcgatctgaaggccatcgtg

C Q H P I V I M R D V N W C D L K A I V

gagttcatgtaccgcggagagatcaacgtgtcccaggaccagattggacccctgctgcgc

E F M Y R G E I N V S Q D Q I G P L L R

attgccgagatgctgaaggtgcgcggactggccgatgtgacccatatggaggccgccgcc

I A E M L K V R G L A D V T H M E A A A

acggccgccgccgccgccgccgccgccgccgccgtggccacagcctcggcctccagcaca

T A A A A A A A A A A V A T A S A S S T

accagtgagcagcagcaggtgtcgagccccaaggagacccagcgcgaggccgccgagcgc

T S E Q Q Q V S S P K E T Q R E A A E R

gaggccgccgaggagctgctggcctttatgcagccggagaagaagctgcgcctgggagcc

E A A E E L L A F M Q P E K K L R L G A

acagattggggcgattacggaggcggaggcgagctgcgcctgtcccccctggagcgcccg

T D W G D Y G G G G E L R L S P L E R P

caggtggcccgcaacgtgcgcaagcgccgctggcccagtgccgatacacccatgccgatc

Q V A R N V R K R R W P S A D T P M P I

ttcaatcccccgtccagtagccccctgtcgagcctgatcgccgccgagcgcctggagcag

F N P P S S S P L S S L I A A E R L E Q

gagcagaaggagcgcgagcgccagcgcgactgcagtctgatgaccccgccaccgaagccg

E Q K E R E R Q R D C S L M T P P P K P

tcgggcgccacgaccccacgccgcctgacggagatccatggactggatatgcccagcccc

S G A T T P R R L T E I H G L D M P S P

gccacgaccccggcccccgccattggactgggccgcagcgcccgcaccctggccccctcc

A T T P A P A I G L G R S A R T L A P S

ccgcagcagcagcatcagcagcagcagcagcagcagcagcagcagcagcagcagcagcag

P Q Q Q H Q Q Q Q Q Q Q Q Q Q Q Q Q Q Q

cagcagcagcagcagctgcagcatagcctgcactcgcatagtcattcgctgcatcacccg

Q Q Q Q Q L Q H S L H S H S H S L H H P

cccccacccccgccacatcccagctcccatccacccccacaccatagccagctgggccat

P P P P P H P S S H P P P H H S Q L G H

agcagtccggcctcgtcccagagtggcgcctcggccgccgtgcaccaggccgcctcctcc

S S P A S S Q S G A S A A V H Q A A S S

gccgccagtagcccagccggaggcgatggacgcttcccactgggcccagccgccgccatg

A A S S P A G G D G R F P L G P A A A M

gccgccgccgccatggagctgagcgccctgggcccaccgacagagccccgcctgccaccc

A A A A M E L S A L G P P T E P R L P P

ccaccgccccaccatcagggcggatcgaccgccagtagctccctggccgatgacatggag

P P P H H Q G G S T A S S S L A D D M E

attaagccaggaattgccgagatgattcgcgaggaggagcgcgccaagatgatggagaat

I K P G I A E M I R E E E R A K M M E N

agccacgcctggatgggcgccaccggcagcacactggccgccgacagttatcagtatcag

S H A W M G A T G S T L A A D S Y Q Y Q

ctgcagagtatgtggcagaagtgctggaacaccaatcagaacctgatgcaccatatgcgc

L Q S M W Q K C W N T N Q N L M H H M R

tttcgcgagcgcggcccgctgaagtcgtggcgcccagagacaatggccgaggccatcttt

F R E R G P L K S W R P E T M A E A I F

agcgtgctgaaggagggcctgtcgctgagccaggccgcccgcaagtatgatattccctac

S V L K E G L S L S Q A A R K Y D I P Y

ccgacattcgtgctgtacgccaaccgcgtgcacaatatgctgggaccatccattgacggc

P T F V L Y A N R V H N M L G P S I D G

ggccccgatctgcgcccaaagggccgcggacgcccgcagcgcatcctgctgggaatctgg

G P D L R P K G R G R P Q R I L L G I W

ccggacgagcacatcaagggcgtgattaagacggtggtgttccgcgatgccacaaaggag

P D E H I K G V I K T V V F R D A T K E

ctgaaggacgattccgccctgggcctgggaggccatatgcccccgtacggacgccatagc

L K D D S A L G L G G H M P P Y G R H S

gatatgtcgctgtcgtatccaggagccgccagcgccgccgccctggcctgcagcaatggc

D M S L S Y P G A A S A A A L A C S N G

atgggcggaggcggcgccggagtgggagtgggcgtgggcggaggcccctccgtggtggga

M G G G G A G V G V G V G G G P S V V G

ggaccgggccccgatcagggccagatgagtcaggagaccgccgccgccgtggccgccgtg

G P G P D Q G Q M S Q E T A A A V A A V

gcccacaatatccgccagcagatgcagatggccgccgccgtgcagcagcagcaccagcac

A H N I R Q Q M Q M A A A V Q Q Q H Q H

ggagaggccggccccccgcccggactgttcaatctgcccccgcacctggccggaagtggc

G E A G P P P G L F N L P P H L A G S G

ccggtgctgggccgcggcagcagtatcagcccggccctgtcctcgggaagtggcccccgc

P V L G R G S S I S P A L S S G S G P R

cacgccccgcccagtagtccctgcggcccagccggcctgatgcccaacctgccgcccagc

H A P P S S P C G P A G L M P N L P P S

atggccgtggccctgcaccgcggcgatcccgccgccgcccaggccctgatctcgcatcag

M A V A L H R G D P A A A Q A L I S H Q

cagcagcagcagcagcagcagcagcagcagcagcatcagcaccacctgcagcagctgcag

Q Q Q Q Q Q Q Q Q Q Q H Q H H L Q Q L Q

cagcagcatcaccaggccctgcagcagcagcagcagcagcagcagcagcagcagcagcag

Q Q H H Q A L Q Q Q Q Q Q Q Q Q Q Q Q Q

cagcaccagcaggccgccgcccaccatggcctgacccacaagagctccggcttcggagcc

Q H Q Q A A A H H G L T H K S S G F G A

agcagtatgccagccgccaacgtggccagttcgtccagtagtcagcagcagcagcagcag

S S M P A A N V A S S S S S Q Q Q Q Q Q

cagcagttccatcagcagctggataagccaaagaccaagggctccccgatgcgcagtgag

Q Q F H Q Q L D K P K T K G S P M R S E

accccacgcctgcattcgcccctgaccgatctgggcctggagatgagctcctacaagcgc

T P R L H S P L T D L G L E M S S Y K R

gattatagcccatcgcgcctgttcgccgatgacctggccgagctggtgggagccggaggc

D Y S P S R L F A D D L A E L V G A G G

ggcggagccgcctccgtgtcctcgagcagcagtgccacaaccacagccgccgccgccgcc

G G A A S V S S S S S A T T T A A A A A

gccgccgccgccgccgccgccgccgccgccgccgccgtggccgccgccacaagtggcaca

A A A A A A A A A A A A V A A A T S G T

tccggctccggaggagacgccagtggaatcaaggtggagcccattacgacaacatcggga

S G S G G D A S G I K V E P I T T T S G

gagtagtaggcggccgc

E - - - -
