# Supplementary material for: Cis-regulatory evolution integrated the Bric-à-brac transcription factors into a novel fruit fly gene regulatory network
Source: eLife. 2018 Jan 3;7:e32273. doi: 10.7554/eLife.32273 (PMC5752203; doi:10.7554/eLife.32273)
Supplement: Transparent reporting form [file elife-32273-transrepform.docx]

***eLife’s* transparent reporting form**

We encourage authors to provide detailed information *within their submission* to facilitate the interpretation and replication of experiments. Authors can upload supporting documentation to indicate the use of appropriate reporting guidelines for health-related research (see [EQUATOR Network](http://www.equator-network.org/%20)), life science research (see the [BioSharing Information Resource](https://biosharing.org/)), or the [ARRIVE guidelines](http://www.plosbiology.org/article/info:doi/10.1371/journal.pbio.1000412) for reporting work involving animal research. Where applicable, authors should refer to any relevant reporting standards documents in this form.

If you have any questions, please consult our Journal Policies and/or contact us: [editorial@elifesciences.org](mailto:editorial@elifesciences.org).

**Sample-size estimation**

- You should state whether an appropriate sample size was computed when the study was being designed
- You should state the statistical method of sample size computation and any required assumptions
- If no explicit power analysis was used, you should describe how you decided what sample (replicate) size (number) to use

Please outline where this information can be found within the submission (e.g., sections or figure legends), or explain why this information doesn’t apply to your submission:

The number of replicate samples were determined based on our past experiences with the experiments utilized here.

**Replicates**

- You should report how often each experiment was performed
- You should include a definition of biological versus technical replication
- The data obtained should be provided and sufficient information should be provided to indicate the number of independent biological and/or technical replicates
- If you encountered any outliers, you should describe how these were handled
- Criteria for exclusion/inclusion of data should be clearly stated
- High-throughput sequence data should be uploaded before submission, with a private link for reviewers provided (these are available from both GEO and ArrayExpress)

Please outline where this information can be found within the submission (e.g., sections or figure legends), or explain why this information doesn’t apply to your submission:

For all experiments presented, we obtained a number of biological replicates tailored to the type of experiment being performed and for which we have found sufficient to drawn robust conclusions from in our past work. For gel shift assays, this refers to performing the same experiment on two different occasions. For experiments with fruit flies, biological replications indicate comparisons of groups of sibling pupae (reporter transgenes and immunohistochemistry) or adult flies (abdomen pigmentation patterns) that were treated at the same time.

The green fluorescent protein expressions shown in Figure 2 panels C-J are biological replicates that are representative of sample sizes of 3, except for panel 2I which we were only able to obtain a sample size of 2. The gel shift assay experiments were done twice, with representative data presented in Figure 3 and Figure 4. The RNA-interferences results shown in Figure 5 are representative samples of 4 biological replicates. Representative specimens from 4 biological replicates are shown for each genotype in Figure 6 where we looked at pigmentation phenotypes following Bab protein ectopic expression. The reporter transgene expressions shown for various genotypes in Figure 7 and Figure 8 are representative specimens from 5 biological replicates, except for Figure 8C which is a representative sample of 4 biological replicates. The representative specimens for the mutant reporter transgene expression shown in Figure 2-figure supplement 1 come from 3 biological replicates. The immunohistochemistry results shown in Figure 6-figure supplement 4 are representative specimens from 3 biological replicates for each genotype.

The discussion of biological replicates for the quantitative reporter transgene assays and pigmentation phenotypes can be found in the “Imaging and analysis of abdomens” section in the materials and methods. No data was treated as an outlier for this study.

**Statistical reporting**

- Statistical analysis methods should be described and justified
- Raw data should be presented in figures whenever informative to do so (typically when N per group is less than 10)
- For each experiment, you should identify the statistical tests used, exact values of N, definitions of center, methods of multiple test correction, and dispersion and precision measures (e.g., mean, median, SD, SEM, confidence intervals; and, for the major substantive results, a measure of effect size (e.g., Pearson's r, Cohen's d)
- Report exact p-values wherever possible alongside the summary statistics and 95% confidence intervals. These should be reported for all key questions and not only when the p-value is less than 0.05.

Please outline where this information can be found within the submission (e.g., sections or figure legends), or explain why this information doesn’t apply to your submission:

The precision measures for quantitatively comparing reporter transgene expression is described in the “Imaging and analysis of abdomens” section in the materials and methods. We used this quantitative comparison method because we had used it successfully for experiments presented in 4 prior publications that we referenced in the methods section. The raw data for pixel intensity measurements and the performed precision measures (mean and SEM) will be provided as “Source Data” excel files that will be uploaded.

(For large datasets, or papers with a very large number of statistical tests, you may upload a single table file with tests, Ns, etc., with reference to sections in the manuscript.)

**Group allocation**

- Indicate how samples were allocated into experimental groups (in the case of clinical studies, please specify allocation to treatment method); if randomization was used, please also state if restricted randomization was applied
- Indicate if masking was used during group allocation, data collection and/or data analysis

Please outline where this information can be found within the submission (e.g., sections or figure legends), or explain why this information doesn’t apply to your submission:

Not applicable here.

**Additional data files (“source data”)**

- We encourage you to upload relevant additional data files, such as numerical data that are represented as a graph in a figure, or as a summary table
- Where provided, these should be in the most useful format, and they can be uploaded as “Source data” files linked to a main figure or table
- Include model definition files including the full list of parameters used
- Include code used for data analysis (e.g., R, MatLab)
- Avoid stating that data files are “available upon request”

Please indicate the figures or tables for which source data files have been provided:

Source data for measurements of pigmentation phenotypes presented in Figure 5 and Figure 6, and quantitative comparisons of reporter transgene expression experiments presented in Figure 7, Figure 8, and Figure 8-figure supplement 1 will be included as excel files.
